# Identification of regulatory network hubs that control lipid metabolism in *Chlamydomonas reinhardtii*

**DOI:** 10.1093/jxb/erv217

**Published:** 2015-05-28

**Authors:** Mahmoud Gargouri, Jeong-Jin Park, F. Omar Holguin, Min-Jeong Kim, Hongxia Wang, Rahul R. Deshpande, Yair Shachar-Hill, Leslie M. Hicks, David R. Gang

**Affiliations:** ^1^Institute of Biological Chemistry, Washington State University, Pullman, WA 99164, USA; ^2^College of Agricultural, Consumer and Environmental Sciences, New Mexico State University, 1780 E. University Ave, Las Cruces, NM 88003, USA; ^3^Donald Danforth Plant Science Center, 975 North Warson Road, St Louis, MO 63132, USA; ^4^ Current address: National Center of Biomedical Analysis, 27 Taiping Road, Beijing, 100850, China; ^5^Department of Plant Biology, Michigan State University, 612 Wilson Road, East Lansing, MI 48864, USA; ^6^Department of Chemistry, University of North Carolina at Chapel Hill, 125 South Road, Chapel Hill, NC 27516, USA

**Keywords:** Biofuel, *Chlamydomonas reinhardtii*, metabolomics, network analysis, proteomics, regulatory hubs, RNA-seq, transcription factors, transcriptional regulators.

## Abstract

Microalgae-based biofuels are promising sources of alternative energy, but improvements throughout the production process are required to establish them as economically feasible. One of the most influential improvements would be a significant increase in lipid yields, which could be achieved by altering the regulation of lipid biosynthesis and accumulation. *Chlamydomonas reinhardtii* accumulates oil (triacylglycerols, TAG) in response to nitrogen (N) deprivation. Although a few important regulatory genes have been identified that are involved in controlling this process, a global understanding of the larger regulatory network has not been developed. In order to uncover this network in this species, a combined omics (transcriptomic, proteomic and metabolomic) analysis was applied to cells grown in a time course experiment after a shift from N-replete to N-depleted conditions. Changes in transcript and protein levels of 414 predicted transcription factors (TFs) and transcriptional regulators (TRs) were monitored relative to other genes. The TF and TR genes were thus classified by two separate measures: up-regulated versus down-regulated and early response versus late response relative to two phases of polar lipid synthesis (before and after TAG biosynthesis initiation). Lipidomic and primary metabolite profiling generated compound accumulation levels that were integrated with the transcript dataset and TF profiling to produce a transcriptional regulatory network. Evaluation of this proposed regulatory network led to the identification of several regulatory hubs that control many aspects of cellular metabolism, from N assimilation and metabolism, to central metabolism, photosynthesis and lipid metabolism.

## Highlight

Characterization of regulatory networks in *Chlamydomonas reinhardtii* led to the identification of regulatory hubs that control the repatterning of cellular metabolism that leads to triacylglycerol accumulation in microalgae.

## Introduction

Microalgae hold great potential as feed stocks for renewable biofuel production and have attracted attention for their ability to biosynthesize large amounts of high-value hydrocarbons while harnessing only sunlight, carbon dioxide and wastewater (Georgianna and Mayfield, 2012). Nevertheless, far more research is needed before algae become commercially viable. Successful rational metabolic engineering of microalgae requires a comprehensive understanding of the regulation of metabolic pathways in the context of the whole cell rather than at the single pathway level (Capell and Christou, 2004). This includes a full understanding of regulatory proteins such as transcription factors (TFs) and transcriptional regulators (TRs), as well as microRNAs, and how they respond to external stimuli and then control downstream processes (Latchman, 1997).

*Chlamydomonas reinhardtii* (Chlamydomonas) is one of the best-studied eukaryotic microalgae, with a known genome sequence and extensive physiological data available. It has been used to investigate a wide range of complex biological processes, including photosynthesis, biomass accumulation, starch metabolism, carbon concentration mechanisms (CCMs) and response to nutrient stress (Ball *et al.*, 1990; Rochaix, 2002; Moellering and Benning, 2010). Nitrogen (N) starvation is among the most stressful conditions that can affect cellular physiology and leads to an increase of neutral lipids (triacylglycerols, TAGs) within a few hours in Chlamydomonas (Fan *et al.*, 2012). Although some genes have been identified to be involved in this response, the underlying sensing and the downstream regulatory mechanisms have not been clearly defined.

With the completion of the Chlamydomonas genome, the entire set of genes encoding members of known TF and TR families can be identified and characterized. However, only a few TFs and TRs have been identified in Chlamydomonas as responding to nutrient stresses. One example is PSR1, a member of the G2-like TF family, involved in regulating the acclimation responses of Chlamydomonas to phosphorus (P) deprivation. Its transcript increases significantly when wild-type cells are exposed to P starvation for 8h (Wykoff *et al.*, 1999). Other examples include the TRs CCM1 (CIA5) and LCR1 (Low CO_2_ Stress Response 1, members of the C2H2-type zinc-finger and MYB-related families, respectively, that are known to regulate CCM activity (Fukuzawa *et al.*, 2001; Miura *et al.*, 2004; Yoshioka *et al.*, 2004). Finally, NRR1, a SQUAMOSA promoter binding domain protein, is the only TF reported to date to be associated with N starvation and lipid accumulation. NRR1 was identified based on its expression pattern relative to a type-1 diacylglycerol acyltransferase (DGTT1) during N starvation (Boyle *et al.*, 2012). More recently and during the preparation of this manuscript, Schmollinger *et al.* (2014) identified two basic helix-loop-helix type transcription factors that were associated with N assimilation under N depleted conditions.

Based on this background, we hypothesized that a correlation network analysis approach (Nikiforova *et al.*, 2005; Allen *et al.*, 2010) could be used to identify proteins that may act in important regulatory roles to respond to external stimuli, such as N deprivation, and then control downstream metabolic outcomes, such as lipid accumulation. Indeed, such an approach, if feasible, should identify the known regulators of N metabolism, such as NRR1. In this investigation, we analysed a correlation network generated using a time course of Chlamydomonas grown under N deprivation, with a focus on the transition in metabolism that occurs when the cells move from the *b*efore *T*AG *s*ynthesis (BTS) phase to the *a*fter *T*AG *s*ynthesis initiation (ATS) phase. More importantly, our objective was not to construct a sparse network but to identify all (or as close to that as possible) key regulators that collaborate to tune lipid synthesis under N stress conditions. To achieve this, we compared the expression of all 414 TFs and TRs predicted in the Chlamydomonas genome to metabolite levels and protein and transcript levels for metabolic enzymes associated with the important biological processes that are active during the two major phases (BTS and ATS) of the response to N deprivation and that ultimately lead to neutral lipid accumulation. Importantly, our analysis matched the dynamic metabolic changes that occur during the phase transition into their own sub-networks without any prior knowledge (i.e., N metabolism change).

## Materials and methods

### Strain, growth conditions, and extraction and analysis of transcripts, proteins and primary metabolites

The cell-wall deficient mutant of *Chlamydomonas reinhardtii* CC-400 cw15 mt+ (called ‘cw15’ throughout) was used as the main cell line for this investigation. Cells were grown under standard mixotrophic conditions (Park *et al.*, 2015) under a time course where samples were collected at 0 (control), 0.5, 1, 2, 4, 6, 12, 24 and 48h after transfer to medium lacking N to initiate N deprivation. Transcript, protein and primary metabolite levels were determined as previously described (Lee and Fiehn, 2008; Mortazavi *et al.*, 2008; Harris, 2009; Alvarez *et al.*, 2011; Wang *et al.*, 2012; He *et al.*, 2014; Juergens *et al.*, 2015; Park *et al.*, 2015), as detailed in the Supplementary Materials and Methods at *JXB* online.

To investigate the function of Tab2 in *Chlamydomonas* during N deprivation, a comparison between the parental strain (CC-125) and the *tab2* mutant (kindly provided from IBPC, France) was performed using growth time courses either in the presence (N replete) or absence (N depleted) of N added to the medium [standard tris-acetate-phosphate (TAP) medium, which included 7.5mM NH_4_Cl and 17.5mM acetate]. Both strains (*tab2* and CC-125) were grown at 25°C in continuous light (70 μm photons m^-2^ s^-1^) in the presence of acetate in liquid cultures under shaking (150rpm). For nitrogen starvation studies, exponential phase (4×10^6^ cells ml^-1^) cultures were centrifuged at 1000 ×*g* for 5min at room temperature, with cell pellets kept and washed twice in TAP either with or without N. Pellets were then resuspended in medium without N and cells were grown under constant light with shaking. Samples for analysis were taken immediately after resuspension (time 0) or periodically during the growth time courses, and were pelleted as outlined above.

### Proteome analysis for *tab2* and wild type comparisons

For proteomic analysis, *Chlamydomonas* strains were harvested by centrifugation at 3 000 ×*g* for 5min at 4°C. Proteins were extracted from 50−100mg of cells as described previously (Wang *et al.*, 2012) and were quantified using the Qubit Protein Assay Kit (Invitrogen, Carlsbad, USA) according to the supplier’s protocol for the Qubit 2.0 fluorometer (Invitrogen). From each sample, 100 µg was digested with trypsin and analysed by an Orbitrap Fusion Tribrid mass spectrometer (Thermo Scientific, Rockford, USA) coupled with an EASY-nLC (Thermo Scientific). The resulting proteomics data were processed and searched using SIEVE 2.1 (Thermo Scientific), and all searches were performed against the Chlamydomonas protein database from Phytozome v. 10.0 (http://phytozome.jgi.doe.gov/pz/portal.html) and NCBI chloroplast (http://www.ncbi.nlm.nih.gov/nuccore/BK000554) and mitochondrion (http://www.ncbi.nlm.nih.gov/nuccore/NC_001638.1) databases (for more details see Supplementary Materials and Methods).

### Lipidomic analysis

Lipidomic analyses for the network experiments were performed on an LTQ linear ion trap FT-ICR mass spectrometer (Thermo, San Jose, CA) equipped with an Advion Triversa Nano Mate (Advion, Ithaca, NY). The Nano Mate was operated in positive and negative mode. The resulting Kendrick mass sorted peak list with its corresponding elemental composition was cross-referenced to formulae found within the structure databases available in the Lipid Maps database (Nature Lipidomic Gateway) and the OxPLDB (http://fiehnlab.ucdavis.edu/staff/kind/Metabolomics/LipidAnalysis). The most abundant ions of the non-oxidized glycerol lipids from each Kendrick series were verified by tandem mass spectrometry. Oxidized glycerol lipids were putatively identified by exact mass.

### Correlation analysis and hub definition

Pearson correlations were determined using the statistical software package R. The transcript, and metabolite abundances, collected at the different time points during N deprivation, with duplicates at least of each, were used to calculate the time-lagged correlation values for pairwise comparisons between metabolites, transcripts and transcripts for TFs/TRs (Walther *et al.*, 2010; Redestig and Costa, 2011) as detailed in the Supplementary Materials and Methods. For this analysis, the fold change was calculated for TFs/TRs, metabolites and metabolic genes/proteins specific for each biological process relative to the time zero (control condition). The criteria for correlation determination were correlation values of ≥0.9 or ≤−0.9 and *P*-values ≤0.05. Graphical visualization of the correlation network was performed using Cytoscape 3.0 (http://www.cytoscape.org/). The data used to build the correlation network were imported into the Cytoscape program as an EXCEL file containing the correlation values as well as a description of the different nodes: TFs/TRs (circles), metabolic genes/proteins (squares) and metabolites (triangles). Each pair of nodes, TF versus metabolic gene or TF versus metabolite with a correlation coefficient ≥0.9 was connected by a line indicating a positive (+1) or negative (-1) correlation and was retained in the network. All network motifs of the same type were merged to construct a motif-specific subnetwork [for example, all SIMs (single input motifs) were merged to form the SIM subnetwork] and then the subnetwork of each biological process was visualized using the Cytoscape software. The hub nodes were defined as the top 5% highest-degree nodes of the TFs in both the subnetwork and whole network.

## Results and discussion

### Expression of TF and TR genes in Chlamydomonas in response to N-depletion

Using the pipeline and basic rules for identification and classification of transcription factors and transcriptional regulators adopted by PlnTFDB 3.0 (http://plntfdb.bio.uni-potsdam.de/v3.0/), we identified in our Chlamydomonas transcriptional profiling data a total of 241 putative TFs that belong to 37 different protein families and 173 putative TRs that are members of 21 families based on the presence or absence of one or more characteristic domains (normally signature DNA-binding domains, see Supplementary Dataset S1). The largest TF and TR families in Chlamydomonas were the GCN5-related-N-acetyltransferase (GNAT) and TRAF (*T*umor Necrosis Factor *r*eceptor-*a*ssociated *f*actor) domain families, respectively, with 36 and 37 members and which accounted for 8.6 and 8.9% of the total number of TF and TR genes detected. The GNAT domain family uses acyl-CoAs to acylate their cognate substrates (Vetting *et al.*, 2005). The TRAF domain family is a relatively uncommon gene family in animal systems, with just one member in *C. elegans*, two in Drosophila and just six in mammals. Why they are so expanded in the unicellular alga Chlamydomonas is unclear at present.

Most transcription factors did not exceed a 2-fold change in transcript abundance across the N-deprivation time course (Supplementary Fig. S1A). Of those that did change significantly in expression (defined conservatively by us as greater than 2-fold change in at least one of the time points relative to control), more TFs and TRs were up-regulated rather than down-regulated after N-depletion (Supplementary Figs S2, S3; and see comparison of *P*-values and false discovery rate [FDR] versus fold change in Supplementary Dataset S1 for all genes used in this analysis). In contrast to the transcriptional profiling data, our proteomics data identified a much smaller set (11.2%) of TFs and TRs as being differentially expressed. This difference was due to the fact that these proteins are typically present at very low copy number per cell since they act as regulatory elements and do not need to be expressed at high levels themselves, as discussed in (Greenbaum *et al.*, 2002). These proteins can be easily masked by highly abundant proteins, making them often difficult to detect in proteomics investigations. That we identified 28 TFs and TRs in the proteomics data (Supplementary Dataset S1) is indicative of the power of the Orbitrap Fusion Tribrid mass spectrometer. Gene ontology analysis indicated that most TFs and TRs in Chlamydomonas are associated with metabolic processes (Supplementary Fig. S1B).

### Establishment of correlation networks to identify transcriptional regulatory candidates

**Fig. 1.**
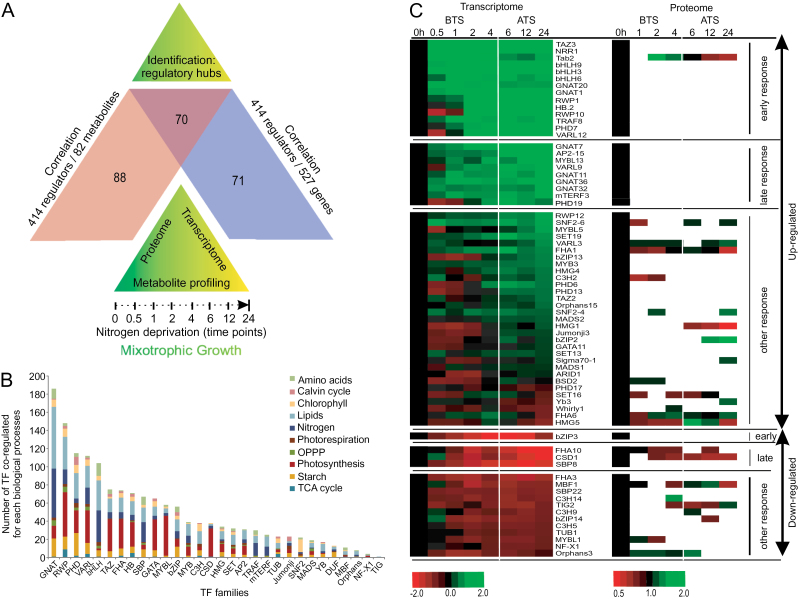
Establishment of correlation networks for identification of transcriptional regulatory candidates. (A) Overview of the experimental design used to identify the correlation regulatory networks in *Chlamydomonas reinhardtii* cw15 grown under N deprivation stress and sampled using different ‘omic’ datasets obtained at multiple time points. (B) Distribution of TF and TR family members that displayed correlations with genes involved in different biological processes and that were differentially expressed in Chlamydomonas during N deprivation. Y-axis values indicate the number of genes from each TF and TR family (indicated on the X-axis) that were highly correlated from different biological processes, indicated by colour. Only those correlations that in absolute value were not smaller than 0.9 were used to generate this figure. See Supplementary Table S3 for details of the specific genes used to generate this graph. (C) Transcriptomic and proteomic profiling of the 70 TFs/TRs that were common to the two sets of correlation analysis, TFs/TRs versus metabolites and TFs/TRs versus genes. Heat maps show the expression level of transcripts and the accumulation of proteins for each identified TF/TR during the N deprivation time course. The transcript expression levels are presented as log base-2 fold change relative to time zero. The protein accumulation values are presented as a ratio relative to 1. The data are classified in two groups: ‘up-regulated’ (green) and ‘down-regulated’ (red). Each group was sub-classified into three sub-groups: ‘early response’, ‘late response’ and ‘unaltered’ based on pattern of expression relative to the onset of the accumulation of TAG. BTS (Before TAG Synthesis) corresponds to the time period 0.5–4h for early responses and ATS (After TAG Synthesis initiation) corresponds to 6–24h for later responses.

To generate the topology of potential transcriptional coexpression networks that are likely to contain genes involved in metabolic regulation and control of TAG accumulation in Chlamydomonas during N deprivation (Fig. 1), we exhaustively analysed co-responses between all TFs and TRs in the genome and the set of 82 primary metabolites that accumulated differentially under these conditions (Supplementary Dataset S1), using an established approach based on time-lag Pearson correlation analysis (Walther *et al.*, 2010; Redestig and Costa, 2011). To ensure maximum specificity and efficiency during this analysis, stringent criteria were used for cut-off values: *R*-value >0.9 and *P*-value <0.05 (Supplementary Dataset S1). Before data were integrated in this analysis, a Shapiro-Wilk’s test (*P*>0.05) (Shapiro and Wilk, 1965) was applied to check the normality of the time series data. A visual inspection of the resulting histograms, normal Q-Q plots and box plots showed that both the transcript and metabolite data were approximately normally distributed, with a skewness of 0.906, 1.232, 1.639, 1.779, 0.662, −0.127 and 0.406 (SE=0.086) and a kurtosis of −0.947, 0.401, −1.482, −1.730, 1.374, −0.313 and 0.790 (SE=0.172) for the corresponding time series (0.5, 1, 2, 4, 6, 12 and 24h) of transcript data, respectively, and a skeweness of −1.46, −0.482, 1.317, 2.005, −0.327, 0.777 and 0.157 (SE=0.369) and a kurtosis of −0.051, 1.128, −0.441, 1.179, −1.479, −1.748 and −0.022 (SE=0.724) for the time series of metabolite data, respectively, (Supplementary Fig. S4) (Doane and Seward, 2011). All of these z-values are within ±1.96, indicating that both transcript and metabolite data are not significantly skewed but are kurtotic, and that the datasets therefore do not differ significantly from normality. Because of that, we concluded that we could use Pearson correlation coefficients to construct the correlation networks described in this study. The metabolite data used for this correlation analysis were for 32 organic acids, 18 fatty acids, 20 amino acids and 12 sugars. Those metabolites that showed differential accumulation over the time course, out of a few hundred compounds that were measured (most compounds were not differentially accumulated), led to the identification of 158 TF genes that were highly correlated with 58 metabolites.

Based on the annotated Phytozome 10 database (Goodstein *et al.*, 2012), 10 sub-networks were generated containing genes involved in lipid metabolism (152 genes), the Calvin-Benson-Bassham cycle (23 genes), N metabolism (67 genes), photosynthesis (85 genes), photorespiration (12 genes), the oxidative pentose phosphate pathway (OPPP, 13 genes), the citrate and glyoxylate cycles (15 genes), sucrose/starch metabolism (64 genes), amino acid metabolism (41 genes) and chlorophyll synthesis (55 genes) (see Supplementary Dataset S1). These were included in the correlation network analysis. Based on this analysis, 141 TF and TR genes belonging to 30 gene families showed strong correlations with lipid metabolism and photosynthesis, suggesting that these latter two processes are highly susceptible to regulation and metabolic readjustments during N deprivation (Fig. 1B). When we compared the two correlation networks (TFs versus metabolites and TFs versus metabolism genes), we found that 70 TFs were in common, being highly correlated with the metabolites as well as their corresponding genes (Fig. 1C). The expression profiles of most of these common TFs could be divided into two major groups: 46 TF genes were up-regulated whereas 16 TFs were down-regulated across the N deprivation time course. In addition, lipidomic analysis for the same set of cultures demonstrated that TAG accumulation was initiated between 4 and 6h after N deprivation (Supplementary Fig. S5). Accordingly, the ‘down-regulated’ and ‘up-regulated’ groups could be further sub-classified as BTS and ATS, as defined above, using the 2-fold change (or *P*-value) as a cut off value (Fig. 1C).

### Association of transcription factors to specific biological processes during N deprivation

In order to identify TFs and TRs that are likely to be involved in the regulation of specific metabolic processes, we analysed three main components of the regulatory network: transcript levels for TF genes, transcript levels for metabolic enzymes, and metabolite levels. As a result, we were able to identify many interesting correlations that suggest how specific TF genes are involved in regulating important metabolic processes in Chlamydomonas that are associated with the transition to lipid accumulation or to the direct response to N deprivation. Each general area of metabolism will be discussed in turn.

#### Nitrogen metabolism

**Fig. 2.**
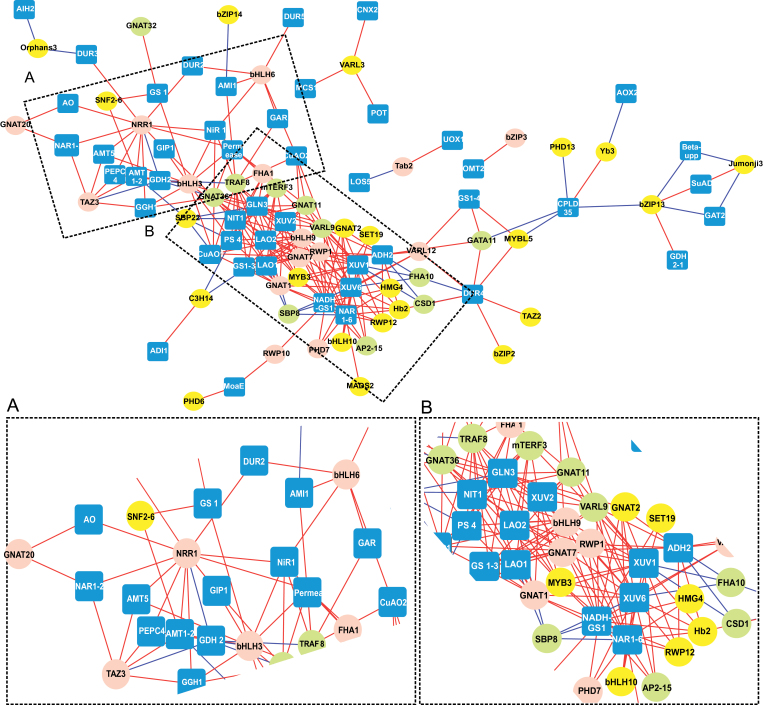
Visualization of the nitrogen metabolism regulatory network in Chlamydomonas during N deprivation that included 67 metabolism-related genes and the 70 TFs/TRs that were differentially expressed. Inset (A): The subnetwork that included TF/TR genes that showed the highest correlations during the early phase (1–4h) with genes involved in the assimilation and transport of inorganic N. Inset (B): The subnetwork that included TF/TR genes that were highly correlated during the later phase (6–24h) with genes required for assimilation of organic N. Nodes: TFs/TRs are represented by pink circles (for the early responders), green circles (for late responders) and yellow circles (for those with a different response). Metabolism-related genes are represented by blue squares. Lines connecting two nodes represent significant correlations: red represents a positive correlation and blue represents a negative correlation. See Supplementary Table S3 for details on genes included in this analysis.

As mentioned in the Introduction, NRR1 (Cre16.g673250) is an N responsive regulatory protein. It is a member of the SBP family and increases (Boyle *et al.*, 2012) in expression along with an ammonium transporter, *AMT1D*, during N starvation. In agreement with those findings, our N metabolism correlation sub-network (Fig. 2A) showed that *NRR1* was not only closely co-expressed with ammonium transporter genes *AMT5*, *AMT1-2* and *AMI1*, but also with several N uptake genes, e.g. *NIR1*, thus supporting the hypothesis that NRR1 is a ‘master’ transcriptional regulator required for reprogramming gene expression of N metabolism during N deprivation, as suggested by (Schmollinger *et al.*, 2014). Interestingly, we also noticed that this regulator was highly correlated with *NAR1-2*, a formate nitrite transporter gene demonstrated to encode a nitrite and bicarbonate transporter in *Xenopus oocytes* that responds to *CCM1*, which is the central regulatory gene for carbon assimilation (Mariscal *et al.*, 2006). Further studies are needed to demonstrate the role of *NRR1* in integrating C and N metabolism during N deprivation.

A second TF associated with N metabolism in our analysis was TAZ3 (Cre03.g212977), which displayed the highest change in expression (>5-fold change) (Fig. 1C and Supplementary Dataset S1) and the earliest response to N deficiency compared to all other potential regulatory genes. *TAZ3* had a similar expression profile to *NRR1* (although increasing in expression prior to *NRR1*) and these two genes appear to be co-regulated within the first 30min after the shift to N-deprived conditions, suggesting that an upstream regulatory protein controls these genes. Alternatively, it is possible that *TAZ3* plays a role in regulating *NRR1*. Future experiments to determine the epistatic relationship of these genes will answer this question.

In Chlamydomonas, the RWP-RK family is involved in the regulation of genes in response to N status (Camargo *et al.*, 2007; Lin and Goodenough, 2007). NIT2, an RWP-RK protein, is a positively acting regulator of the nitrate assimilation pathway and its transcript levels increased 6-fold after 48h of N deprivation (Miller *et al.*, 2010). In our study, two RWP family members, *RWP1* (Cre10.g453500) and *RWP10* (Cre03.g149350), responded early to N starvation (by 2h) (Supplementary Dataset S1). Their transcripts increased across the time course up to >3-fold and were correlated with the expression of glutamine synthetase (*GS*) and NADH-dependent glutamate synthase (*NADH-GS*). As seen in Fig. 2, *RWP10* was also highly correlated with a molybdopterin biosynthesis enzyme (*MoaE1*, Cre10.g451400). In plants, molybdenum cofactors are important for nitrate assimilation and purine catabolism. A mutation in molybdenum cofactor biosynthesis leads to the combined loss of nitrate reductase activity and assimilation of inorganic N (Mendel and Hänsch, 2002). This indicates that RWP10 may regulate molybdopterin activity for the assimilation of nitrate early in response to N deprivation. On the other hand, *RWP1* was positively correlated with two periplasmic L-amino acid oxidases, *LAO1* and *LAO2* (Fig. 2B). These enzymes catalyse the deamination of all L-amino acids and participate in the assimilation of extracellular amino acids. While no external amino acids were added in our experiment, *RWP1* may be induced as a general response to N depletion in case such compounds may be available in the environment. In addition, members of the bHLH and GNAT TF families (*bHLH3, bHLH6* and *GNAT20*) were coexpressed with the N-metabolism genes during the first 4h (Fig. 2A). Taken together, these data indicate that at least five TF/TR families (SBP, TAZ, bHLH, GNAT and RWP-RK) may be involved in regulating genes required for assimilation and transport of any inorganic N source (ammonium, nitrite and nitrate) available during the early phase, as well as the incorporation of ammonium into carbon skeletons via the glutamine synthetase/glutamate synthase (GS/GOGAT) cycle.

Once TAG had begun to accumulate within the cells, *FHA10*, *CSD1* and *SBP8* transcript levels were down-regulated and negatively correlated with xanthine/uracil permeases (*XUV1* and *XUV6*), genes involved in the purine degradation pathway (Fig. 2). Chlamydomonas cells use purines as a source of organic N during TAG accumulation (Schmollinger *et al.*, 2014). These combined results suggest that *FHA10*, *CSD1* and *SBP8* may be suppressors of purine catabolism under N replete conditions. In contrast, *GNAT11*, *GNAT36*, *VARL9* and *TRAF8* are positively correlated with two copper-containing amine oxidases (*CuAO1* and *CuAO2*) that oxidize putrescine for the production of NH_3_ (Fig. 2). These results suggest that six TF families (FHA, CSD, SBP, GNAT, VARL and TRAF) are involved during the ATS phase in an attempt to compensate for lack of external free N by regulating purine catabolism.

#### Photosynthesis

Several TF genes were found to be correlated with photosynthesis-related genes, such as light harvesting and electron transfer components, as well as Calvin-Benson-Bassham cycle enzymes. *NRR1* and *TAZ3* were the only TFs correlated with *PSBS* and *LHCSR1* (Supplementary Fig. S6A). PSBS plays a critical role in non-photochemical quenching (NPQ) and is induced by N deprivation (Miller *et al.*, 2010). LHCSR, which is member of the LHC (light harvesting complex) superfamily, plays an important role in response to high light and protection against photo damage (Peers *et al.*, 2009). The abundance of LHCSR protein increases as NPQ increases during N-deprivation (Schmollinger *et al.*, 2014). Thus, NRR1 and TAZ3 appear to be involved during the BTS phase of N deprivation to optimize photosynthetic function and minimize photo-oxidative damage.

*VARL12* (Cre14.617200), another early responding TF gene, was negatively correlated with expression levels of several photosynthetic genes. Its level of expression doubled by 4h, reaching its highest change (~3.6-fold) at 24h (Supplementary Dataset S1). The VARL family has not been characterized functionally but it contains a DNA binding SAND domain. Another member of this gene family, *VARL7* (also called *RSL1*), is up-regulated when Chlamydomonas cells are grown heterotrophically in the dark, suggesting that it may be a regulator of photosynthetic gene expression (Nedelcu and Michod, 2006). An additional TF gene, *PHD7* (Cre10.g446600), was correlated with cytochrome b6f subunits, such as PetO, PetM and PetN, but *RWP1*, *RWP10*, *GNAT1* (Cre06.g278108) and *GNAT7* (Cre05.g236900), were mostly co-expressed with core subunits of photosystem I (PSI), photosystem II (PSII) and ATPase (Supplementary Fig. S6). Thus, the regulation of photosynthesis that was observed by others during the early phase of N deprivation (Schmollinger *et al.*, 2014) appears to be multifactorial with members of several TF families, such as SBP, TAZ, PHD, RWP-RK, GNAT and VARL, involved in the adjustment of light energy utilization under the high stress conditions of N depletion.

A different set of TF genes appeared to play later roles in remodelling photosynthesis. *FHA10*, *CSD1* and *SBP8* were positively correlated with several photosynthesis genes during the ATS phase (Supplementary Fig. S6). CSD1, a cytosolic RNA-binding protein, preferentially binds to *LHCM6* mRNA (Mussgnug *et al.*, 2005; Wobbe *et al.*, 2009) and plays an important role in controlling the expression of the light-harvesting antenna of PSII at the post-transcriptional level. FHA10, on the other hand, contains a forkhead transcription factor domain, a class of proteins that play a role in the DNA-damage response as well as mediating interactions with proteins phosphorylated by serine/threonine kinases (Durocher and Jackson, 2002). FHA10 and CSD1 may be activators of photosynthesis under N replete conditions. A member of the MYB family, *MYBL13* (Cre01.g034350), also responded late to N limitation. Its transcript levels increased progressively to a 2.6-fold change by 12h (Supplementary Dataset S1) and it was positively co-expressed with several photosynthetic genes. MYBL13 harbours a SANT domain, which is mainly found in proteins involved in chromatin remodelling (Boyer *et al.*, 2002) and interacts with histone N-terminal domains (Boyer *et al.*, 2004). In addition, an AP2/EREBP family member, *AP2-15* (Cre16.g667900), was positively correlated with *MYBL13*. This is not surprising since the MYB and AP2/EREBP families are involved in the regulation of photosynthesis and related metabolism under stress conditions (Vannini *et al.*, 2004; Karaba *et al.*, 2007; Saibo *et al.*, 2009).

Several Calvin-Benson-Bassham cycle genes were significantly down-regulated during N deprivation, and specific TF genes were found to be potential regulators of those enzymes. *VARL12, PHD7* and *RWP10* were positively correlated with phosphoglycerate kinase (*PGK1*), which in turn was negatively correlated with an additional member of the PHD-finger family, *PHD19* (Cre08.g358543, Supplementary Fig. S6B). PHD proteins are involved in controlling chromatin structure (Bienz, 2006), suggesting a possible mechanism for regulating the abundance of Calvin-Benson-Bassham cycle enzymes. Chlamydomonas bZIP13 (Cre01.g051150) shares 30% identity with Long Hypocotyl 5 (HY5) from higher plants, a bZIP-type protein known to be involved in the regulation of expression of chlorophyll a/b-binding protein, as well as the transcription of other photosynthesis-related genes, such as ribulose bisphosphate carboxylase small subunit during abiotic stress (Lee *et al.*, 2007). In Chlamydomonas, however, *bZIP13* was negatively correlated with several photosynthesis genes (Supplementary Fig. S6), including ribulose-1,5-bisphosphate carboxylase/oxygenase small subunit 2 (*RBCS2*) (Supplementary Fig. S6B). Together these observations suggest that four TF families, VARL, PHD, RWP-RK and bZIP, may participate in the down-regulation of genes involved in light absorption and carbon fixation and are involved in controlling the C/N ratio during the ATS phase of N deprivation.

#### Chlorophyll metabolism

In Chlamydomonas, the decline in photosynthetic yield during N deprivation is followed by a rapid decline of chlorophyll concentration (Schmollinger *et al.*, 2014). It has been suggested that chlorophyll degradation increases following N deprivation to remobilize N from associated proteins and to reduce the light stress (Geider *et al.*, 1998). Chlorophyll synthesis genes for both the porphyrin ring and phytol chain were rapidly down-regulated, with transcript abundance falling by one half of initial values by 2h, while transcript abundances for chlorophyll catabolic genes, such as chlorophyllase (*Chlase*), were significantly elevated after 1h. *RWP1*, *PHD7*, *HB2*, *bHLH9*, *GNAT1* and *GNAT7* were negatively correlated with key genes in the methylerythritol-phosphate pathway (*HDR* and *CMK*) and in chlorophyll formation (*ACL1*, *MgPP* and *PPO1*) (Czarnecki and Grimm, 2012), but they were positively correlated with *Chlase* and zeaxanthin epoxidase (*ZEO*), enzymes required for carotenoid synthesis (Supplementary Fig. S6C). Carotenoids are involved in light absorption and energy dissipation, reducing radical oxygen species production. Within the first 4h of N deprivation, the expression of some carotenoid synthesis genes was strongly up-regulated while the expression of most such genes stayed at the same level or decreased. After 4h, most of the transcripts related to carotenoid synthesis decreased, while those encoding the degradation enzymes carotenoid cleavage dioxygenases 1 and 2 were elevated. Together, these observations suggest that the TF genes listed above probably contribute to regulating the protection of Chlamydomonas cells against photo-oxidative stress induced by N deprivation via the up-regulation of chlorophyll degradation and carotenoid synthesis. These observations are in agreement with an increase of free zeaxanthin levels during the first 4h of N deprivation (Baroli *et al.*, 2003; Juergens *et al.*, 2015), which likely contributes to protection of Chlamydomonas cells against photo-oxidative stress induced by N deprivation (Li *et al.*, 2012b). Interestingly, transcript levels of carotenoid isomerase (*CRI1*) were correlated with *bHLH3* and with *GNAT32* (Supplementary Fig. S6C). In higher plants, CRI1 is required for the formation of prolamellar bodies (PLBs), which accelerate photomorphogenesis. PLBs may reflect the stable presence of the protochlorophyllide oxidoreductase-protochlorophyllide complex in plants that have lost the capacity to synthesize chlorophyll in the dark (Sperling *et al.*, 1998). This suggests that GNAT32 and bHLH3 constitute a regulatory complex in Chlamydomonas that controls photomorphogenesis by regulating CRI1 expression levels during N deprivation.

#### Photorespiration

Photorespiration serves as a carbon recovery system (Maurino and Peterhansel, 2010) and is an important mechanism to keep the PSII repair system functional under stressful conditions (Takahashi *et al.*, 2007). Most genes associated with photorespiration were rapidly up-regulated by 1h of N deprivation. At 4h, the expression of serine hydroxymethyltransferases 2 and 3 (SHMT2 and SHMT3), enzymes catalysing the reversible reaction of L-serine to glycine, was even more enhanced, while most other photorespiration genes stayed at the same level or decreased compared to the 1h time point and thus were still elevated compared to N replete conditions. Among the TFs responding early to N deprivation, two bHLH family members (*bHLH3* and *bHLH9*, Supplementary Fig. S6D) were positively correlated with phosphoglycolate phosphatase (*PGP3*) and cytosolic hydroxypyruvate reductase (*HPR2*), respectively. Also, *Tab2* and *TAZ3* were positively correlated with glycerate kinase (*GYK1*), an enzyme involved in the last step of 3-PGA formation from glycerate. On the other hand, among the TFs responding late to N deprivation *AP2-15* was positively correlated with serine accumulation (Supplementary Fig. S6E), and *PHD19* and *GNAT32* were positively correlated with SHMT 2 and 3 transcripts (Supplementary Fig. S6D). These results suggest that even though regulation of photorespiration may be complex in Chlamydomonas, several distinct TFs appear to be involved and thereby contribute to protection of PSII during the ATS phase.

#### Oxidative pentose phosphate pathway (OPPP)

The OPPP plays a critical role in providing reducing equivalents (NADPH) to cells that lack the capacity to generate sufficient reducing power via a fully functional PSI. The OPPP is a major source of reductant for fatty-acid synthesis and assimilation of inorganic N. It is also required to maintain the proper cellular redox state under oxidative stress conditions (Kruger and von Schaewen, 2003). Several transcription factors, including *bHLH9*, *VARL12*, *GNAT7* and *mTERF3* (Cre09.g408050), were positively correlated with 6-phosphogluconate dehydrogenase (*6PGD*) and glucose-6-phosphate dehydrogenase (*G6PD*) (Supplementary Fig. S6E). These two enzymes are the steps in the OPPP where the reducing equivalents are formed. Reduced N availability would decrease photosynthetic activity and affect the NADPH/ATP ratio. Thus, the levels for enzymes involved in the OPPP appear to be regulated under N deprivation to maintain the proper redox state under this stressful condition. However, this could dramatically affect photosynthetic performance and yield during N deprivation. Elucidation of the exact role of specific enzymes in the OPPP under these conditions will require more focused and targeted efforts than were possible in this study.

#### Carbohydrate metabolism

Tab2 (Cre17.g702500) is an RNA-binding protein belonging to the DUF (domain of unknown function) group of proteins that is localized in the chloroplast stroma where it is associated with a high molecular mass protein complex containing *PsaB* mRNA. Dauvillée *et al.* (2003) proposed that Tab2 plays a key role in the initial steps of PsaB translation and PSI assembly. *Tab2* mRNA levels increased ~2.5-fold after 30min and its protein level was also significantly increased after 2h of N deprivation (Fig. 1C). Surprisingly, this potential regulatory protein showed low correlation with genes involved in photosynthesis, whereas it was highly correlated with a number of transcripts encoding glycolytic enzymes, such as phosphoglucomutases (PGM1 and PGM2), glucose-6-phosphate isomerase (PGI) and pyruvate kinase (PYK2), as well as with the metabolites G6P and fructose-6-phosphate (F6P) (Supplementary Fig. S7A). These results suggest that Tab2 executes during the BTS phase of N deprivation a novel or a combinatorial function related to the regulation of genes directly involved in carbohydrate metabolism. Work that identifies how Tab2 does this will be presented in a separate manuscript.

The accumulation of fructose and glucose was observed at and after 6h of N deprivation (Supplementary Fig. S6). PHD19 was positively correlated with the accumulation of fructose, invertase (INV1) and alpha-amylase (AMA3), whereas bZIP13 was correlated with fructose, G1P, INV2 and phosphofructokinase (PFK2), but was negatively correlated with three isoforms of glucose-1-phosphate adenyltransferase (GLGS1, GLGS2 and GLGS3), which catalyse an initial step in starch production (Supplementary Fig. S6A). These results indicate that PHD19 and bZIP13 may be involved in regulating the switch from the gluconeogenic state to a glycolytic state (Park *et al.*, 2015) that occurs prior to initiation of lipid accumulation in Chlamydomonas in response to N deprivation.

#### Citrate and glyoxylate cycles

The citrate cycle is initially down-regulated in Chlamydomonas within the first few hours after N depletion but is then upregulated by 24h (Blaby *et al.*, 2013; Park *et al.*, 2015). Several TF genes identified in this investigation, including *RWP1*, *HB2* and *VARL12*, showed a strong negative correlation with the citrate cycle genes, isocitrate lyase (*ICL1*) and malate synthase (MAS1) (Supplementary Fig. S6F). Acetate was added to the medium to provide a carbon source for the cells. This externally supplied acetate is assimilated to form acetyl-CoA, which then feeds into the glyoxylate and citrate cycles and is further used for gluconeogenesis and rapid starch storage before TAG accumulation initiates (Fan *et al.*, 2012). The action of regulatory proteins controlling this process fits well with the model developed (see below) for the transition to lipid accumulation following N deprivation.

#### Amino acid metabolism

Relative abundance data for the 20 standard amino acids and 41 transcripts encoding known enzymes involved in their biosynthesis were compared to the 70 TF genes identified as responding to N deprivation (Supplementary Fig. S6E). The resulting correlation network pointed to several TF genes that appeared to have strong connections to important components of amino acid metabolism. Some of these amino acid pathways are intimately connected to other central metabolic processes, such as photorespiration, N metabolism, and the citrate and glyoxylate cycles and were discussed above. Two other examples stood out as particularly interesting.

The first involves the interesting situation of L-arginine (L-Arg) biosynthesis in N-deprived Chlamydomonas cells. One TF of interest with regards to L-Arg metabolism was *bHLH6*, which displayed a positive correlation with PII (pii), *N*-acetyl-gamma-glutamyl-phosphate reductase (NAGPR), N-acetyl-L-glutamate kinase (NAGK) and arginosuccinate synthase (AsuS), all components of arginine metabolism, but a negative correlation with the metabolites L-Arg and L-Orn. *MYBL13* was also positively correlated with two isozymes of carbamoyl-phosphate synthase (Lc CPS and Sc CPS), while at the same time being negatively correlated with glutamine levels. These enzymes catalyse the ATP-dependent synthesis of carbamoyl phosphate from glutamine as the entry point into arginine biosynthesis. These results suggest that bHLH6 and MYBL13 are involved in regulating arginine levels under N deprivation.

An additional TF that may play an important role in regulating amino acid metabolism in response to N deprivation was bZIP14 (Cre16.g653300), which displayed a close positive correlation with genes involved in L-histidine (L-His) biosynthesis, such as histidinol dehydrogenase (*HDH*) and imidazole glycerol-phosphate dehydratase (*H5B*), as well as with the accumulation of histidine. In fungi, the regulation of L-His biosynthesis is tightly coordinated with purine biosynthesis by a bZIP transcription factor, GCN4p (Springer *et al.*, 1996). Treatment of *Arabidopsis* with imidazole glycerol-phosphate dehydratase inhibitor (IRL1803) led to up-regulation of imidazole glycerol-phosphate synthase 8 and purine biosynthesis (Guyer *et al.*, 1995). These results suggest that bZIP14 may down-regulate L-His biosynthesis, allowing for preferential reassimilation of N into purines during the later stages of N deprivation, at the expense of L-His levels (Schmollinger *et al.*, 2014).

#### Lipid metabolism

Little is known about transcriptional regulation of lipid biosynthesis in Chlamydomonas. Previous studies seeking to identify the role of putative transcription factors or other regulatory proteins in regulation of lipid metabolism assessed only expression profiles of target genes at a single time point. Moreover, most of the previous reports describe cells evaluated once TAG levels are high, after two days of N deprivation or later (Miller *et al.*, 2010; Lv *et al.*, 2013). As indicated in more recent work, the repatterning of metabolism that follows the switch to N depleted conditions and leads eventually to TAG accumulation begins almost immediately after the growth medium is changed (Park *et al.*, 2015), indicating that the cells recognize and start to respond to the change in environment within minutes, not hours or days. Our correlation analysis approach provided an opportunity to identify TFs that may be involved in regulating lipid repatterning following the switch to N depleted medium based on their transcript or protein levels relative to the expression profiles of other genes during the time course of N deprivation, beginning from the onset of nutrient removal from the media through initiation of lipid accumulation.

**Fig. 3.**
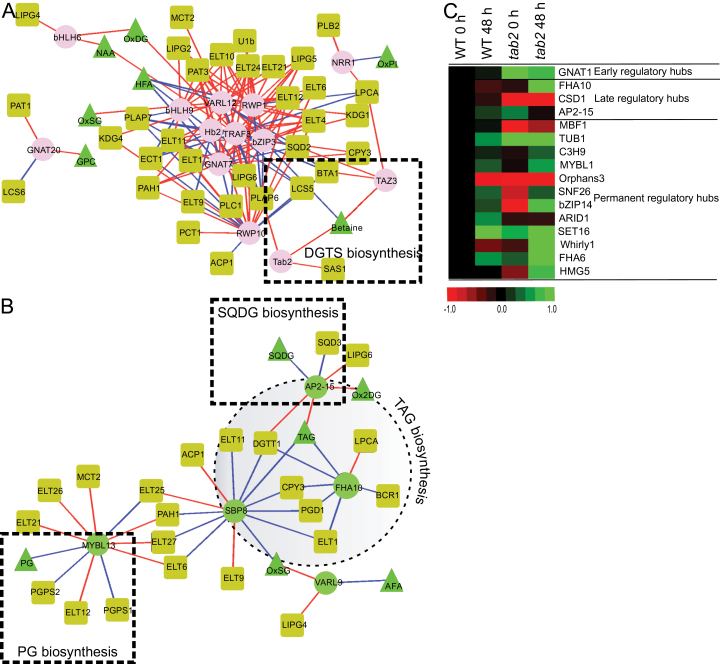
Visualization of the lipid metabolism regulatory networks in Chlamydomonas during N deprivation that included 152 lipid metabolism-related genes and the 70 TFs/TRs that were differentially expressed. (A) The subnetwork that included TF/TR genes that showed the highest correlations during the BTS phase (0.5–4h) with many lipases and a subset of genes involved in membrane lipid biosynthesis. (B) The subnetwork that included TF/TR genes that were highly correlated during the ATS phase (6–24h) with TAG and its related biosynthetic genes. Nodes: TFs/TRs as in Fig. 2. Metabolism-related genes are represented by olive-coloured squares. Lines indicating significance as in Fig. 2. See Supplementary Dataset S1 and Supplementary Fig. S6 for details on genes included in this analysis. (C) Visualization of protein expression levels of regulatory hubs in WT after 48h of N deprivation, *tab2* mutant at time 0 and *tab2* mutant after 48h of N deprivation, all relative to WT at time 0.

As discussed above, several TF genes were identified as being early responders to N deprivation, and several of these are likely to play important roles in regulating lipid accumulation and repatterning. The TF NRR1, was originally identified based on a supposed correlation with DGTT1 (Cre16.g673250) expression, and the *nrr1* mutant displayed a reduction in the accumulation of TAG following N deprivation (Boyle *et al.*, 2012). However, we found that *NRR1* did not display a high correlation with *DGTT1* in our study (*R* value was <0.6), which included a more detailed time course of N deprivation. Instead, *NRR1* was positively correlated with the *PLB2* gene, encoding a putative phospholipase B-like protein (Fig. 3A). Our RNA-seq analysis indicated an increase in *PLB2* transcript levels (>2.5-fold after 1h) (Supplementary Dataset S1) that matched the pattern displayed by *NRR1*. Many other putative lipase-encoding genes showed increases in their mRNA abundances early on, and were also highly correlated with early responding TFs. Only five lipases (*PAT1*, *ELT1*, *ELT4*, *ELT10* and *ELT 24*) decreased in abundance at the early time points (Supplementary Dataset S1). Among the most highly induced during the early phase were *LIPG2*, *LIPG4*, *LIPG5*, *LIPG6* (putative triacylglycerol lipases), *PAT3* (patatin), *ELT6*, *ELT9*, *ELT12* and *ELT21* (esterase/lipase family), and *PLC1* (coding for phospholipase C). The levels of transcripts for all of these lipases increased in abundance by only 30min of N deprivation and were positively correlated with two members of the bHLH family (bHLH6 and bHLH9), two members of the GNAT family (GNAT7 and GNAT20), two members of the RWP family (RWP1 and RWP10), bZIP3, HB2, TRAF8 and VARL12 (Fig. 3A). In addition, these TFs were positively correlated with oxidized membrane lipids (oxidized phosphatidylinositol, oxidized MGDG), acylated sterol glucoside and 1-alkyl,2-acylglycerophosphocholines and negatively correlated with the accumulation of hydroxyl-fatty acids. This correlation pattern goes hand in hand with the accumulation levels of these metabolites during the first 6h following N deprivation (Supplementary Fig. S5) during the BTS phase, and suggests that these early transcription factors are involved in the large regulatory network that controls remodelling of lipid membranes under N deprivation. N deprivation-induced lipase genes might be involved in shuffling fatty acids from the membrane lipids to TAG. For example, an enzyme capable of cleaving FAs from MGDG has been identified in Chlamydomonas (Li *et al.*, 2012a) providing a possible mechanism for recycling of this glycolipid. Based on our data, we would suggest that similar enzymes also exist for the liberation of FAs from DGDG and SQDG. Thus, FAs derived from the breakdown of SQDG could be used in TAG synthesis. However, to compensate for the loss of the chloroplast glycolipids, Chlamydomonas and many other microalga appear to up-regulate PG synthesis (Martin *et al.*, 2014).

*Tab2* and *TAZ3* were positively correlated with the levels of betaine (trimethylhomoserine, Supplementary Fig. S6), as well as with *S*-adenosylmethionine synthetase (*SAS1*) and betaine lipid synthase (*BTA1*) mRNAs, respectively (Fig. 3A). These two enzymes are involved in diacylglyceryltrimethylhomoserine (DGTS) biosynthesis and were up-regulated during the first hour of N deprivation, followed by a progressive decrease in expression across the rest of the time course (Supplementary Dataset S1). DGTS is a major class of membrane lipids in Chlamydomonas. The pattern of rapid increase and then a steady decrease in DGTS content is consistent with the remodelling of the lipid profile that occurs in Chlamydomonas during the BTS phase as the cells prepare to accumulate TAG to high levels.

Other TF genes responded later to N deprivation, and thus play roles in later phases of cellular remodelling during the ATS phase. The expression of AP2-15 (Cre16.g667900, a member of the AP2/EREBP family) remained relatively constant during the first 2h and then noticeably increased through 24h, reaching a 2.5-fold change (Fig. 1C) overall. AP2-15 is one of the 17 AP2 TFs in Chlamydomonas with a high similarity (E-value of 1×10^–42^) to rice, maize and *Arabidopsis* AP2-domain containing proteins (Supplementary Fig. S8A). Due to its similarity to maize WRINKLED 1 (WRI1), it could be called CreWRI1-like. *AP2-15*’s expression pattern was highly correlated with an increase in total fatty acid levels in response to N-limitation. This result mirrors the function of WRI1 and similar proteins in vegetative tissues of several plants (Baud *et al.*, 2007; Bourgis *et al.*, 2011). Furthermore, members of the AP2/EREBP family may be involved in integration of signals derived from organelles in retrograde feedback loops and in stress acclimation (Dietz *et al.*, 2010). This TF was positively correlated with increasing amounts of TAG (Supplementary Fig. S6) and DGTT1, which exhibited increasing transcript levels after 2h and reached a maximum after 24h of N deprivation (Supplementary Dataset S1). It is interesting to consider that a similar role in integration of stress signals may be found for AP2-15 in Chlamydomonas as was found for its higher plant counterparts. In contrast to what was observed relative to TAG biosynthesis, AP2-15 was negatively correlated with a component of plastidic membranes, the sulfolipid sulfoquinovosyldiacylglycerol (SQDG), and the corresponding biosynthetic enzyme, sulfoquinovosyldiacylglycerol synthase (SQD3) (Fig. 3B). SQDG levels decreased beginning after 6h of N deprivation (during the ATS phase, Supplementary Fig. S6). Thus, AP2-15 may play a role in reprogramming the cell’s lipid composition by controlling the TAG/photosynthetic membrane lipid ratio during the ATS phase of N deprivation. Similarly, transcripts for one member of the FHA TF family, *FHA6* (Cre16.g671900), were up-regulated over the 24h time course, and its protein levels were significantly elevated by 2h following N deprivation (Supplementary Dataset S1). *FHA6*, like *AP2-15*, was negatively correlated with the decreasing levels of SQDG and *SQD2*, suggesting that it too might be a suppressor of SQDG biosynthesis during N deprivation (Supplementary Fig. S8B).

In contrast to AP2-15, the TFs FHA10 and SPB8 were negatively correlated with the accumulation of TAG, DGTT1 and Plastid Galactoglycerolipid Degradation1 (PGD1), see Fig. 3B. PGD1, an MGDG-specific lipase, acts predominantly on more saturated forms of MDGD, specifically removing 16:0 and 18:1Δ9 from newly synthesized MGDG. This enzyme may be involved in re-shuffling saturated FAs from MGDG to TAG in a non-Kennedy pathway route to TAG synthesis (Li *et al.*, 2012a). The mRNA levels of *PGD1* increased progressively after 2h of N deprivation (Supplementary Dataset S1). Interestingly, these TFs (FHA10 and SPB8) were also negatively correlated with *ELT1* (Cre01.g000300), which codes for an alpha/beta hydrolase family protein that has high similarity to the Thaps3_264297 protein (E-value of 1.3×10^–46^). THAPS protein homologues to CGI-58 in *Arabidopsis* have been proposed to be involved in lipid breakdown, leading to increased TAG yield without affecting growth in the diatom *Thalassiosira pseudonana* (James *et al.*, 2010; Trentacoste *et al.*, 2013). As indicated above, we observed that these TFs were positively correlated with many photosynthetic genes. One possible explanation for this finding is that FHA10 and SBP8 function as suppressors of TAG biosynthesis genes and activators of photosynthesis genes. Taken together, these findings suggest diverse and independent functions for distinct TAG lipases during the two phases, which is consistent with the variable expression levels of lipases seen in the transcriptomic analysis (Supplementary Dataset S1), and demonstrates the importance of performing the proper time-course analyses in guiding targeted manipulations.

Interestingly, *MYBL13* was negatively correlated with phosphatidylglycerophosphate (PG) and two genes encoding phosphatidylglycerophosphate synthases (PGPS1 and PGPS2, Fig. 3B) and dropped in expression immediately after the cells were transferred to N deficient medium (by 0.5h). It remained down-regulated for the entire 24h time course (Supplementary Dataset S1). However, PG levels remained stable during the 24h and strongly decreased only at 48h (Supplementary Fig. S6). Because the cells are still dividing during the first 24h and require PG for the synthesis of membrane lipids, the up-regulation of MYBL13 suggests that it might be involved in regulating the redirection of FAs from PG to TAG biosynthesis.

The transcript levels of *bZIP2* (Cre07.g321550) fluctuated slightly, but otherwise remained fairly constant (Supplementary Dataset S1). However, the proteomic data clearly showed that the protein levels increased significantly from 12h to 24h (Fig. 1C). Thus, bZIP2 was positively correlated with increasing TAG levels and *DGTT1* (Supplementary Fig. S6B), suggesting that bZIP2 may play a role in regulating TAG accumulation, perhaps by affecting *DGTT1* expression. Indeed, in higher plants, the bZIP TF family regulates several processes including light response, stress signalling, seed maturation, flower development, cell elongation, C/N balance, hormone and sugar signalling, and seed storage protein gene regulation (Corrêa *et al.*, 2008). However, Chlamydomonas bZIP TFs have not been characterized functionally, and the only sequences found in the non-redundant database of NCBI (http://www.ncbi.nlm.nih.gov/) with significant homology to these proteins correspond to hypothetical proteins from *Volvox carteri f. nagariensis* (E-value of 5×10^–16^). There is no evidence for an orthologue in land plants for bZIP2. Further functional characterization of this candidate regulatory gene is necessary to elucidate its regulatory roles in N stress response and TAG accumulation.

The TR, *SET13* (Cre17.g742700), was also positively correlated with increasing TAG levels, DGTT1 mRNA levels, and the transcript levels of the Major Lipid Droplet Protein (MLDP1) (see Supplementary Fig. S8B). MLDP is a major protein associated with lipid droplet formation and its repression affects lipid droplet size but not TAG levels (Moellering and Benning, 2010). SET13 showed high similarity to SET DOMAIN GROUP 2 (SDG2; E-value of 1×10^–56^), a transcriptional regulator that plays a distinctive role in the regulation of chromatin structure and genome integrity during root growth and development in *Arabidopsis* (Yao *et al.*, 2013). It is thus possible that SET13 in Chlamydomonas contributes to TAG regulation by affecting expression of MLDP.

### Identification of transcriptional regulatory hubs that control lipid accumulation

Complex networks have underlying architectures guided by universal principles. For instance, many networks, from the World Wide Web to the cell’s metabolic system to airport connections, are dominated by a small number of nodes that are highly connected to other nodes. These important nodes, called hubs, greatly affect the network’s overall behaviour. These hubs make the network robust against accidental failures but vulnerable to coordinated attacks. As outlined below, the results of this investigation demonstrate the presence of such hubs in the metabolic and regulatory networks that control lipid accumulation in algal systems such as Chlamydomonas.

As observed above, analysis of the transcriptional regulatory networks found that some TFs are involved in multiple sub-networks, suggesting a hierarchical structure whereby specific TFs might play major synergistic roles in the greater regulatory network, and may therefore function as hubs. To identify specific TFs that might function as hubs, we analysed the degree of distribution of nodes in the 10 sub-networks present in a combined network (Supplementary Fig. S1C). Most nodes had low degrees and only a few nodes had high degrees of distribution, reflecting a scale-free network structure and indicating the presence of few highly influential TFs (regulatory hubs) that regulate expression of several genes and a large number of TFs that regulate a few genes and confer a robustness to the network (Babu, 2008). Analysis of time-course expression changes of TFs at the level of network structure and their interconnectivity in different biological processes allowed for the identification of two major types of hubs, specific hubs and permanent hubs, which represent a highly robust and flexible core within the regulatory network that controls the transitions associated with alga growth during the shift from nutrient-replete to -depleted conditions. While the permanent hubs are those TFs that affect expression of several genes independent of the perturbation condition, the condition-specific hubs regulate genes during specific cellular conditions. BTS- and ATS-specific hubs were identified as TFs that regulate the expression of several target genes in different biological processes (single input motif) during the BTS or ATS phases. These network motifs, for the BTS- and ATS-specific hubs, have temporal patterns that are unique compared to the permanent hubs. The list of regulatory hubs is shown in Supplementary Table S1. The top five, with the highest degrees of centrality, are shown in Table 1. Thus, these specific hubs represent critical components of two very different regulatory modules, one that sets the stage for initiation of lipid synthesis (BTS module) and a second (ATS module) that carries out the regulatory program that was established earlier and insures that the cell is able to cope long term with the change in metabolic patterning and programming that was triggered by the switch to N deprived medium, several hours earlier.

**Table 1.** *The top five regulatory hubs for specific phases in the response of* Chlamydomonas *to N deprivation*

	**BTS specific hubs**					**ATS specific hubs**					**Permanent hubs**				
Metabolic pathway regulated	VARL12	HB2	GNAT1	bHLH9	GNAT7	FHA10	CSD1	AP2-15	MYBL13	SBP8	RWP1	RWP10	TAZ2	MYBL5	GATA11
Photosynthesis	•	•	•			•	•	•	•		•	•	•	•	•
Nitrogen	•			•	•	•	•	•		•	•	•	•	•	
Chlorophyll biosynthesis		•	•	•	•	•	•				•	•	•	•	•
Photorespiration	•	•		•	•			•		•	•	•	•		
OPPP	•	•	•	•	•					•	•	•			
Calvin cycle	•	•		•	•			•		•	•	•	•		
Carbohydrates	•	•		•		•			•		•	•	•		
Central metabolism	•	•		•	•	•	•				•	•			•
Amino acids	•	•	•	•		•	•	•	•	•	•	•		•	•
Lipids	•	•		•	•	•		•	•	•	•	•	•		•
Degree of centrality	67	74	25	40	45	56	46	26	25	32	60	55	59	36	64

To verify that the putative regulatory hubs are involved in directing the cellular response to accumulate TAG during N deprivation, we analysed a mutant Chlamydomonas line, where Tab2, a highly connected node and an early, BTS-specific hub (Supplementary Table S1), was reduced in expression (Dauvillée *et al.*, 2003), and accumulated 50% of the TAG that wild-type cells produce under N stress conditions (Supplementary Fig. S9). As shown in Supplementary Figs S10 and S11, quantitative real-time PCR demonstrated that the transcript levels of PGM1, PGM2, PKY2, DGTT1 and PDAT1, involved in carbohydrate and lipid metabolism, were differentially affected in the *tab2* mutant compared to the wild type during N deprivation conditions. Proteomic analysis demonstrated that the protein levels of the BTS-specific hub, *GNAT1* (increased 1.66-fold), and the ATS-specific hubs, *FHA10* (increased 2.81-fold), *CSD1* (decreased 0.56-fold) and *AP2-15* (decreased 0.8-fold), were significantly altered in the *tab2* mutant compared to the wild type after 48h of N deprivation (Fig. 3C). However, several permanent regulatory hubs did not show significant changes in protein levels between the wild type and mutant during N deprivation. These results suggest that the *tab2* mutation affects specifically the regulators of the condition-specific hubs, which results in collapse of the network into small sets of isolated fragments that no longer interact with each other (Albert *et al.*, 2000), reflected physiologically by a dramatic decrease of TAG production. Because Tab2 has been reported to be a key component in the initial steps of PsaB translation and photosystem I assembly, one of the plausible explanations for this network behaviour is that the *tab2* mutation disrupts the photosynthesis sub-network that affects specifically the expression of the condition-specific hubs such as FHA10, CSD1 and AP2-15, which then could be involved in the regulation of different sub-networks. This is an issue that needs to be addressed in future research. Nevertheless, the highly connected proteins (hubs) listed in Table 1 are crucial for maintaining the robustness and proper function of the regulatory network that allows for TAG accumulation during N deprivation.

### A regulatory model for N deprivation response and TAG accumulation in Chlamydomonas

**Fig. 4.**
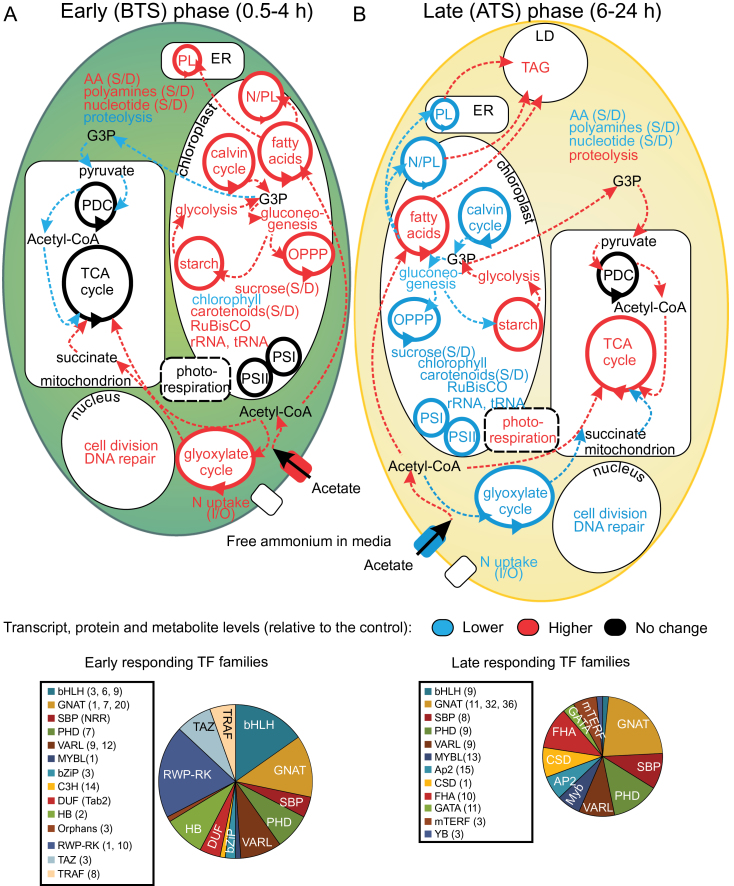
Model of how Chlamydomonas responds to N deprivation (A) during the BTS phase (0.5–4h) and (B) ATS phase (6–24h). Pathways highlighted in red contain genes, proteins and metabolites significantly up-regulated; those in blue contain genes, proteins and metabolites significantly down-regulated; and those in black do not display significant change. Abbreviations: S/D, ratio of synthesis/degradation; I/O, ratio of inorganic/organic N; LD, lipid droplets; PL, polar lipids; N/PL, non- and polar lipids; ER, endoplasmic reticulum; PDC, pyruvate dehydrogenase complex; PSI, photosystem I; PSII, photosystem II. The association of putative transcription factors (TFs) with each state is depicted in pie charts; sizes are proportional to the total number of correlations detected. TFs that were significantly enriched under those conditions are listed in two boxes, with abbreviations defined in the text or in Supplementary Dataset S1.

Based on the analysis presented herein, about 70 TF and TR genes were found to be differentially expressed and were able to be incorporated into correlation networks, which shed new light on regulation of metabolism and cellular growth in response to N deprivation as well as the TAG accumulation that follows. The combined results increase our understanding of the chronological regulatory changes that occur before and after TAG accumulation initiates and allow us to propose a schematic model for the transcriptional regulatory cascade during N deprivation in Chlamydomonas (Fig. 4). According to this model, very early response TRs sense and respond to N deprivation by activating BTS-specific hubs that down-regulate chlorophyll biosynthesis and by increasing expression of genes involved in N assimilation, acetate assimilation, the Calvin-Benson-Bassham cycle, starch and sugar alcohol accumulation, OPPP metabolism and remodelling of lipid membranes. These early responding TF and TR genes include those involved in chromatin remodelling, nucleosome displacement, and alteration of RNA stability, reinforcing the hypothesis that the early responding regulatory genes are establishing a short-term acclimatization to what could be a quickly reversed stress (Fig. 4A). When the N stress is prolonged, dramatic metabolic changes and/or the BTS phase TRs induce a limited number of novel transcription family members, which appear to execute more specific functions related to the induction of genes directly involved in TAG metabolism and formation of lipid droplets, this includes *AP2-15*, *FHA10* and *MYBL13* (Fig. 4B).

All of these results suggest that TAG metabolism is under tight transcriptional control during N deprivation. Moreover, many of the early responding TF genes appeared time and again in our analysis as correlating well with various metabolic processes, supporting their roles as regulatory hubs. Two groups of hubs, specific hubs and permanent hubs, were identified and build a highly robust and flexible core within the regulatory network that controls the transitions associated with algal growth and lipid synthesis after the shift from nutrient-replete to -depleted conditions. Such knowledge will enable synthetic biology approaches to alter the response to the N depletion stress and lead to rewiring of the regulatory networks so that lipid accumulation could be turned on in the absence of N deprivation, allowing for the development of algal production strains with highly enhanced lipid accumulation profiles. Beyond providing insights into the regulatory systems-level organization of Chlamydomonas metabolism during N deprivation, this dataset and approach sets the stage for an emerging series of studies that will decipher the dynamic regulatory networks in other microalgae.

## Supplementary material

Supplementary data are available at *JXB* online.

Supplementary Materials and Methods S1.

Supplementary Dataset S1. Expression levels and annotation of the genes involved in various metabolic processes, including transcription factors and transcriptional regulators, used for the correlation analysis to compare against metabolite levels.

Supplementary Table S1. List of the regulatory hubs identified during N deprivation.

Supplementary Figure S1. The degree of distribution of nodes in the 10 sub-networks present in a combined network indicates a scale-free network structure and the presence of few highly influential TFs.

Supplementary Figure S2. Heat map showed that TFs were up-regulated rather than down-regulated after N-depletion.

Supplementary Figure S3. Heat map showed that TRs were up-regulated rather than down-regulated after N-depletion.

Supplementary Figure S4. Tests of normality for transcript and metabolite data.

Supplementary Figure S5. Primary metabolite profiles during the N deprivation time course.

Supplementary Figure S6. Cytoscape visualization of correlation networks for different biological processes.

Supplementary Figure S7. Visualization of the amino acid biosynthesis and the starch/gluconeogenesis/glycolysis regulatory networks during N deprivation.

Supplementary Figure S8. Visualization of the entire lipid metabolism regulatory network during N deprivation and the tree of the Chlamydomonas AP2-EREBP TF gene family.

Supplementary Figure S9. Changes in TAG content over the time course in the wild-type and *tab2* mutant cells grown in N depleted medium.

Supplementary Figure S10. Verification of gene expression analysis by quantitative real-time PCR.

Supplementary Figure S11. Quantitative real-time PCR validation of some transcript levels in WT and *tab2* cells grown under N deprivation conditions.

Supplementary Data

## Abbreviations:

TAG: triacyglycerol
TF: transcription factors
TR: transcription regulators.
