# Supplementary material for: Identification of regulatory network hubs that control lipid metabolism in Chlamydomonas reinhardtii
Source: J Exp Bot. 2015 May 28;66(15):4551–66. doi: 10.1093/jxb/erv217 (PMC4507760; doi:10.1093/jxb/erv217)
Supplement: Supplementary Data [file supp_66_15_4551__index.html]

Identification of regulatory network hubs that control lipid metabolism in Chlamydomonas reinhardtii — Supplementary Data 

# Identification of regulatory network hubs that control lipid metabolism in *Chlamydomonas reinhardtii*

## Supplementary Data

Data files

- Supplementary Data - Supplementary Data
- Supplementary Data - Supplementary Data
