# Supplementary material for: Identification of regulatory network hubs that control lipid metabolism in Chlamydomonas reinhardtii
Source: J Exp Bot. 2015 May 28;66(15):4551–66. doi: 10.1093/jxb/erv217 (PMC4507760; doi:10.1093/jxb/erv217)
Supplement: Supplementary Data [file supp_erv217_erv217_SuppFigsTable.pdf]

Supplementary online material

Identification of regulatory network hubs that control lipid metabolism in *Chlamydomonas reinhardtii*

Mahmoud Gargouri, Jeong-Jin Park, F. Omar Holguin, Min-Jeong Kim, Hongxia Wang, Rahul Deshpande, Yair Shachar-Hill, Leslie M. Hicks and David R. Gang

This Supplementary online material includes:

Supplementary materials and methods.

Figures: S1, S2, S3, S4, S5, S6, S7, S8, S9, S10 and S11.

Table S1.

Dataset S1.

Supplementary materials and methods.

Strain, media, sampling and culture conditions

The cell-wall deficient mutant of *Chlamydomonas reinhardtii* CC-400 cw15 mt$^+$, was obtained from the Chlamydomonas Resource Center at the University of Minnesota (St. Paul, MN, USA). The cells were cultured in Tris acetate phosphate (TAP) medium (Harris, 2009) containing 5 mM ammonium chloride as N source at 22 °C under constant illumination with cool-white fluorescent bulbs at an illumination of 80 μmol photons·m^{-2}·s^{-1} and with continuous shaking at 150 rpm. Single colonies were used to inoculate a pre-culture in a shake flask plugged with a sponge to allow aeration. This culture was harvested at mid-exponential phase and used to inoculate a new culture at a starting density of around 0.1 OD_{750}, which corresponds to a cell dry weight of 0.05 mg·ml^{-1}. The cells were cultured continuously in a volume of 1 L in a 5 L conical flask by serial dilution, not allowing the cell density to exceed 0.4 OD_{750}. The cells were adapted to this environment by at least three serial dilutions corresponding to more than 5 doublings.

After the cells had been adapted to the above conditions, the culture was then split in two and harvested by centrifugation (5000 ×g for 2 minutes). One half of the centrifuged cells were

washed with acetate-free TAP media and re-suspended in 2 L of fresh TAP media (containing acetate). The remaining cells were washed with acetate free Tris phosphate media without nitrogen source (N-) and re-suspended in 2 L TAP media (with acetate, without N). The cells from each treatment were divided into eight 1 L conical flasks with a culture volume of 200 mL and then allowed to grow under the same conditions described above. The remaining 200 mL of the culture was divided into four 50 mL conical tubes and centrifuged immediately at 5000 ×g for 2 min at 0 ºC for the zero hour time point. Four of the centrifuged pellets were flash frozen in liquid nitrogen and stored at -70 ºC. The total time from harvest to freezing took less than 10 minutes. One of the stored pellets was used for metabolite analysis and the two others were shipped on dry ice for proteomic and lipidomic analysis. To the fourth pellet, 1 mL of the RNA stabilization reagent, RNAlater (Ambion Life Technologies), was added and then frozen in liquid nitrogen for storage at -70 ºC. This sample was used for RNA-seq analysis. The flasks were harvested successively after 0.5, 1, 2, 4, 6, 12 and 24 h for transcriptome and metabolome analysis. However, to accommodate the 4plex iTRAQ experiment design for proteomic analysis, 0.5 h samples were omitted due to slow protein changes. Since TAGs have a delayed accumulation, the centrifuged pellets from the 48 h time point were used only for lipidomic analysis.

RNA extraction and transcriptome analysis

RNA was extracted from *C. reinhardtii* cells preserved in RNAlater using TRIzol reagent (Invitrogen, Karlsruhe, Germany) according to the manufacturer's protocol. The transcriptome analysis was performed as previously described by He et al (He *et al*, 2014). RNA-seq libraries for 51 bp single-read (SR) sequencing were prepared with the TruSeq RNA Sample Prep Kit v1 (Illumina, San Diego, CA) according to the manufacturer's instructions. The sequencing libraries were sent to Macrogen, Inc. (Seoul, Korea) and sequenced on a HiSeq 2000 instrument. Initial base calling and quality filtering of the Illumina SR image data were performed using the Illumina pipeline CASAVA v1.8.2. The raw sequencing reads were trimmed based on a quality value ≥ 20. The trimmed reads were mapped on the *Chlamydomonas reinhardtii* gene coding sequences (the Phytozome ver. 10.0) using CLC Genomics Workbench 5.0 (CLC bio, Aarhus, Denmark). The mapping was performed based on the minimal length of 32 bp with an allowance

of up to two mismatches. The relative transcript abundance was measured in reads per kilobase of exon per million mapped sequence reads (RPKM) (Mortazavi *et al*, 2008).

Protein extraction and proteome analysis

Proteins were extracted from 50–100 mg of cells and 100 μg of each sample was digested with trypsin as described previously (Wang *et al*, 2012). After trypsin digestion, 100 μg of each sample was used for iTRAQ labeling using 4plex iTRAQ reagents according to the manufacturer's instructions (AB Sciex, Foster City, USA). Since there are seven time points in this study (0, 1, 2, 4, 6, 12 and 24 h), two 4-plex iTRAQ experiments were performed for each biological replicate. The samples from 0 h were labeled with reagent 114 in both experiments. The samples from 1, 2 and 4 h were labeled with reagents 115, 116 and 117 in one experiment, respectively, and similarly, the samples from 6, 12, and 24 h were labeled with reagents 115, 116, and 117 in a second experiment, respectively. Labeling was performed as described previously (Wang *et al*, 2012). After labeling, samples from each separate experiment containing labels 114-117 were mixed and fractionated via SCX as described previously (Alvarez *et al*, 2011). Approximately 5% (~18 μg) of the mixture was collected every minute, dried down and cleaned up with a C18 spin column (ThermoFisher Scientific, Rockford, USA) according to the manufacturer's instructions. Individual fractions were then lyophilized and dissolved in 10 μL of 5% ACN, 0.1% formic acid. Samples from 4 to 28 min were analyzed by nano-LC-MS/MS as described previously (Wang *et al*, 2012).

Mass spectral data were processed using Mascot Distiller v2.4 and searched using Mascot Daemon (Matrix Science, London, UK) as described previously (Wang *et al*, 2012) with some modifications. All searches were performed against the merged Chlamydomonas protein database containing sequences from Phytozome and the NCBI chloroplast and mitochondrion databases (19,603 total sequences for the combined dataset) using the same parameters as before.

Extraction and analysis of primary metabolites

Primary metabolites were extracted and analyzed following a previously validated procedure (Lee and Fiehn, 2008). Samples were frozen with liquid nitrogen when they were collected. All samples were stored at -80 °C until required for GC-MS analysis. As biological replicates, samples were collected from 3 individual cultures.

Primary metabolites were extracted from lyophilized samples as follows. Lyophilized samples in 2 ml safe-lock microtubes (Eppendorf) were disrupted using a single 5 mm i.d. steel ball, followed by the addition of 1.0 mL extraction solvent of methanol:isopropanol:water (5:2:2) and vortexing. After 2 min centrifugation at 16,100×g, the supernatants were collected and concentrated to dryness. The dried extracts were reconstituted with a secondary extraction solvent (acetonitrile: water, 1:1). The supernatants were dried for further analysis followed by 5 min centrifugation at 16,100×g. The residue was resuspended in 5 µl of a solution of 20 $mg \cdot ml^{-1}$ of 98% pure methoxyamine hydrochloride (Sigma, St. Louis, MO) in pyridine and shaken at 30 °C for 90 min to protect aldehyde and ketone groups. Next, 45 µl of MSTFA with 1% TMCS (1 ml bottles, Pierce, Rockford IL) was added and shaken at 37 °C for 30 min for trimethylsilylation of acidic protons.

Primary metabolites were separated, identified and quantified on an Agilent 7890 gas chromatograph (Santa Clara, CA) coupled to a time of flight mass spectrometer (Pegasus 4D, LECO, St. Joseph, MI) that had been equipped with an MPS-2 Prepstation sample robot (Gerstel, Muehlheim, Germany) and that used an RTX®-5Sil MS with Integra-Guard® column (30 m × 0.25 mm ID × 0.25 µm film thickness) from Restek (GmbH, Bad Homburg, Germany). The oven temperature was held initially at 50 °C for 1.0 min, raised at 20 $°C \cdot min^{-1}$ to 330 °C, and held for 5.0 min. The column flow (constant) was 1 mL $He \cdot min^{-1}$. A sample volume of 1.0 µL was injected in splitless mode. Mass spectra were acquired from *m/z* 35–500 at a rate of 17 $spectra \cdot s^{-1}$. Primary metabolite data analysis was performed using ChromaTOF software version 4.41, which supports automatic deconvolution of all mass spectra from a chromatogram, built in mass-spectral correction for co-eluting metabolites, calculation of retention indices, and identification of a suitable fragment mass-to charge ratio for selective quantification.

An in-house metabolite mass spectral library and the LECO/Fiehn Metabolomics library were used for identification of compounds. Each identified metabolite was assigned a similarity

value, which is a measurement of the similarity between the collected spectrum and the library mass spectrum. Identifications were only assigned if this value was higher than 700 as a cut-off value. In the Statistical Compare feature of ChromaTOF, the processed samples were added to a sample table and assigned to their respective groups. The alignment processing method provided two parameters, for retention time (RT) and for mass spectral matching. RT match criteria took into account a maximum RT difference and a maximum number of modulation periods between peaks. For spectral matching, a mass threshold and a minimum similarity match were defined. Besides a separate signal to noise ratio for peaks not found by the initial peak finding, thresholds for analytes to be kept for statistical evaluation (minimum number of samples or minimum percent of samples in a class that contain the analyte) were defined.

A log2 fold change of each time point relative to time zero (control condition) with a cut-off value of ± 0.5 was calculated to provide a rough estimate of differential accumulation of metabolites over the time course. This was followed up by using Student's t-test to determine statistical significance (p value < 0.05) of difference relative to control (time 0) for all metabolites at all time points (which matched the 2-fold cutoff estimate). This led to the determination that 82 of the ~150 metabolites that were quantified changed significantly at at least one time point relative to the control.

Functional annotation

For mapping Chlamydomonas genes onto the Kyoto encyclopedia of genes and genomes (KEGG) pathway annotation, we used an annotation DB for gene number with KEGG orthology from the Phytozome website. The Phytozome peptide DB was used for Blast2GO V2.5.0 analysis (Conesa A *et al*, 2005). BLAST was performed using the following settings: blast DB: nr; number of blast hits: 20; blast E cutoff: 1.0×e-3; blast program: BLASTP; blast mode: QBlast-NCBI; high-scoring segment pair (HSP) length cutoff: 33; low complexity filter: on. A total of 17,414 sequences were imported into Blast2GO for BLAST analysis. Annotation was performed with an E-value hit filter of 1×e-6, annotation cut-off of 55, GO weight of 5 and HSP hit coverage of 30. To provide a general representation of the distribution of GO annotation, the GO Slim classification for Plants was obtained, and curated manually.

Quantitative RT-PCR conditions and analysis

Total RNA was isolated from the cell pellet using the Trizol reagent (Ambion). One microgram of total RNA was used as a template for each reverse transcription reaction following the manufacturer's instructions kit (qScript cDNA SuperMix, Quanta Bioscinces). Gene-specific primers were designed to amplify fragments of approximately 100–150 bp in length. For the quantification of gene expression, qPCR was carried out on Mastercycle Realplex 2 (Eppendorf) using the Perfecta Syber Green Fast Mix (Quanta Bioscinces, Gaithersburg, MD). The actin gene served as internal control for the quantification assays in *C. reinhardtii*. For gene expression analysis by qPCR, the expression values were calculated according to the 2-ΔΔCT method (for each gene, ΔCT = CT,Gene − CT,housekeeping specific). The ΔΔCt calculation was validated using the plot of the log cDNA dilution versus ΔCt. See Fig. S11 for all primer sequences used in this work.

Accession Number

Sequences data from this article concerning the time-course N deficiency are deposited in the sequence read archive (http://sra.dnanexus.com) under accession number SRA115617.

Correlation analysis

We performed a time lagged-correlation analysis, where the time-series data were detrended using Minitab 15 statistical software and normalized against the standard deviation before the correlation analysis. The temporal profile of a metabolite, M, and a transcript, T (gene or Transcription factor), can be correlated concurrently, thus pairing up all available time points, t, directly to compute the Pearson correlation coefficient between all [M(t), T(t)]. A high correlation would be an indication of concurrent and potentially coordinated behavior between a particular transcript-metabolite or gene-transcription factor (transcript-transcript) pair. Profile data are paired taking into consideration a time-lag or time-delay. For example, a metabolite may correlated with a transcript, but with a temporal shift to a later time point, t + i, forming [M(t + i), T(t)] pairs. It is clear that upon introducing a time lag, the available time series are shortened, as the (t + i)th data point is not available at the end of the time series and no valid data pair can be

formed. The larger the time delay, the fewer the remaining pairs. As a consequence, the statistical power decreases and a bias toward increased correlation values is introduced by the lower numbers of available data pairs when temporal shifts are considered. Because our available time series consisted of seven data points only (0.5h, 1, 2, 4, 6, 12, 24) we restricted our analyses to a time shift of one time point only, i = 1, that is, transcript or metabolite data were associated with the respective other variable (metabolite or transcript) at the next, successive time point. A much more extensive data set would be required to enable a larger time shift to be employed. A calculation of the FDR was used to threshold the level of significance for the metabolite by gene or gene by gene correlations (Benjamini and Hochberg, 1995). The resulting lag Pearson correlation (LPC) is estimated by modifying the equation of (Redestig and Costa, 2011) as follows:

$$LPC({x_T}^{(l)}, {y_M}^{(l)}) = \max_{l=[-t/2],\ldots,[t/2]} \frac{\sum_{t=1}^{n}({x_{T,t}}^{(l)} - \mu_{x_T})({y_{M,t}}^{(l)} - \mu_{y_M})}{(n-1)S_{x_T}S_{y_M})}.$$

Network motif identification

A preliminary a study was performed using FANMOD (Wernicke and Rasche, 2006), which reports subgraphs that occur significantly more often than in random networks. A network motif was defined based on the criterion that the number of occurrences must be at least five, and also must be significantly higher than that used in randomized networks. The significance test was carried out on 1000 randomized networks, and a pattern with $p < 0.05$ was considered statistically significant. The NetMatch (Ferro et al; 2007) plug-in in Cytoscape was also used to identify single enriched network motifs.

Supplementary Figure Legends

Fig. S1. (A) Distribution of changes in transcription factor (TF) and transcriptional regulator (TR) gene expression in cw15 throughout the time course of N deprivation. Values indicate the number of genes that changed by a certain level (log base 2 scale) at any time point during the time course relative to control (time 0). Only the maximum change in expression level per gene was included in the data used to generate this figure. (B) Transcription factor and transcriptional regulator proteins of Chlamydomonas clustered by GO term (biological process category). Functional Class is the GO term and the GO accession numbers are given. Note that most of the transcriptional regulators have been associated with metabolic processes. (C) Node degree distribution of the synergistic regulatory network (10 sub-networks). The X axis is the degree of a node (number of connections between selected TFs and genes of different biological processes), and the Y axis shows the number of nodes that correspond to the degree.

Fig. S2. Transcription factor expression profiles in cw15 during the N deprivation time course. Gene expression at different time points is presented as fold change (log base 2 scale) relative to the control. The data were visualized as heat maps with green = up-regulated and red = down-regulated.

Fig. S3. Transcriptional regulator expression profiles in cw15 during the N deprivation time course. Gene expression at different time points is presented as fold change (log base 2 scale) relative to the control. The data were visualized as heat maps with green = up-regulated and red = down-regulated.

Fig. S4. Tests of normality for the time series of the transcript and metabolite data. Shapiro-Wilk's values, histograms, normal Q-Q plots and box plots are presented for each time point/each data.

Fig. S5. Primary metabolite profiles in Chlamydomonas during the N deprivation time course. Time points presented for amino acids, organic acids, sugars and sugars alcohol are 0, 0.5, 1, 2, 4, 6, 12 and 24 h. Time points presented for fatty acids are 0, 6, 12, 24, 48 h. Values presented are means of three replicates. OxPI: oxidized phosphatidylinositol; OxDG: oxidized MGDG;

OxSG: Acylated sterol glucoside; GPC: 1-alkyl,2-acylglycerophosphocholines; HFA: hydroxy fatty acids; TAG:triacylglycerol; SQDG: sulfoquinovosyldiacylglycerol; PG: phosphatidylglycerolphosphate; OxTAG: oxidized triacylglycerol; OxPA: oxidized phosphatidic acid; NAA: N-acyl amines; MAB: mono-acyl betaine; Ox2DG: oxidized MGDG/MGDG; MGDG: monogalactosyldiacylglycerol; DAG: diacylglycerol; DGDG: digalactosyldiacylglycerol; AFA: amino fatty acids.

Fig. S6. Cytoscape visualization of correlation networks for different biological processes. (A) photosynthesis, (B) chlorophyll synthesis and degradation, (C) Calvin cycle, (D) photorespiration, (E) oxidative pentose phosphate pathway, (F) citrate and glyoxylate metabolism. Lines connecting two nodes represent significant correlations: red represents a positive correlation and blue represents a negative correlation. See Dataset S3 for details on genes included in this analysis.

Fig. S7. (A) Visualization of the amino acid biosynthesis regulatory network, based on correlation of a subset of 41 genes with the selected TFs/TRs, in Chlamydomonas during 24 h of N deprivation. Nodes: TFs are represented as red circles for early phase responders, green circles for late phase responders and yellow circles for other pattern of response. Genes for metabolic enzymes are represented as pink squares. Amino acids are represented as orange triangles. Lines connecting two nodes represent significant correlations: red represents positive correlation and blue represents negative correlation. See Table S3 for details on genes included in this analysis. (B) Visualization of the starch/gluconeognesis/glycolysis regulatory network, based on correlation of a subset of 64 genes and 12 metabolites with the selected TFs/TRs, in Chlamydomonas during 24 h of N deprivation. Genes involved in starch biosynthesis are represented as lilac squares; genes involved in gluconeogenesis are represented as orange squares; genes involved in glycolysis are represented as gray squares. Metabolites are represented as green triangles. Lines connecting two nodes represent significant correlations: red represents a positive correlation and blue represents a negative correlation. See dataset1 for details on genes included in this analysis

Fig. S8. (A) Neighbor-Joining tree of the Chlamydomonas AP2-EREBP TF gene family, with additional genes with known function included. The similarity tree suggests a close relationship between the AP2-15 (CreWRI-like, cre16.g667900) sequence to that of maize WRI1 and the Arabidopsis relative. Other Chlamydomonas sequences included in this comparison contain a double AP2 domain. The tree was built from multiple alignments of protein sequences, using ClustalW and treeView software. The bar indicates the divergence between the sequences.
(B) Visualization of the entire lipid metabolism regulatory network in Chlamydomonas during N deprivation that included 152 lipid metabolism related genes and the 70 TFs/TRs that were differentially expressed. Nodes: TFs/TRs are represented by pink circles (for the early responders) and green circles (for late responders). Metabolism-related genes are represented by olive colored squares. Lines connecting two nodes represent significant correlations: red represents a positive correlation and blue represents a negative correlation.

Fig. S9. Changes in TAG content over the time course in the wild type and *tab2* mutant cells grown in N depleted medium. Values are averages of triplicate biological samples, ±SE.

Fig. S10. Verification of gene expression analysis by quantitative real time PCR (qRT-PCR). Individual gene expression ratios (nitrogen deprivation sample to control 0 h sample) were calculated using RPKM data generated by RNA-Seq and plotted against calculations done for the same gene using qRT-PCR)

Fig. S11. Verification of gene expression in WT and tab2 cells grown in the N deprivation time course by quantitative real-time PCR (qRT-PCR) analysis. The relative transcript levels (within each graph) are represented by white bars for the WT and black bars for the mutant. The time points displayed are 0.5, 2, 6, 24, 72 hours after N depletion. The box to the right contains the sequences of the primers used in the qRT-PCR analysis for each gene.

Supplementary Table Legend

Table S1. List of the regulatory hubs identified in Chlamydomonas during N deprivation.

Supplementary Dataset legend

Dataset S1. This excel workbook contains 16 worksheets that contain the expression data for all TF, TR and metabolic enzyme genes and proteins discussed in this manuscript. The first three sheets contain the transcript and protein levels of transcription factors (TFs) and transcriptional regulators (TRs) identified under nitrogen starvation. The first sheet lists the log2-fold change of transcript levels for transcription factors (TFs). The second sheet lists the log2-fold change of transcript levels for transcriptional regulators (TRs). And the third sheet compares protein levels to transcript levels for those TRs or TFs that were identified in the proteomics data.

The next three sheets contain the correlation values (Pearson correlation) that were calculated for all expressed TFs (417) and (82) metabolites [(32) Organic acids, (18) Fatty acids, (20) Amino acids, (12) sugars)]. The sheet titled "R values" contains the Correlation values. The sheet called "P values" contains the corresponding p-values for each comparison. The sheet titled "Correlation lists" shows the set of highly correlated TFs to metabolites or transcripts from metabolism genes using a cut-off > |0.9|.

The last series of 10 sheets contains the expression levels and annotation of the genes for specific biological processes used for the correlation analysis, including: nitrogen metabolism, photosynthesis, chlorophyll metabolism, Calvin-Benson-Bassham cycle, photorespiration, OPPP, TCA and glyoxylate cycles, amino acid metabolism, sucrose and starch metabolism, and lipid metabolism.

Supplementary table

Table S1. List of the regulatory hubs identified in Chlamydomonas during N deprivation.

	Photosynthesis	Nitrogen	Chlorophyll	Photorespiration	OPPP	Calvin cycle	Carbohydrates	Central metabolism	Amino acids	Lipids
Early specific hubs										
Cre14g617200t13(VARL12)	•	•		•	•	•	•	•	•	•
Cre16g673250t13(NRR)	•	•					•		•	•
Cre08g375400t13(HB2)	•		•	•	•	•	•	•	•	•
Cre10g446600t12(PHD7)	•		•	•	•	•	•		•	
g6251t2(GNAT1)	•		•		•				•	
g4643t1(bHLH9)		•	•	•	•	•	•	•	•	•
Cre01g011150t13(bHLH3)		•	•	•	•	•	•		•	
Cre05g236900t13(GNAT7)		•	•	•	•	•		•		•
Cre17g702500t12(Tab2)		•	•	•		•	•			•
Cre03g151050t13(TRAF8)		•	•		•		•		•	•
g8224t1(bHLH6)		•	•						•	•
Cre13g568350t13(bZIP3)		•							•	•
Cre14g612100t13(RWP12)					•	•	•		•	
Late specific hubs										
Cre10g418600t13(VARL9)	•	•	•	•	•	•			•	•
g7712t1(GNAT36)	•	•	•		•		•		•	
Cre12g534450t13(FHA10)	•	•	•				•	•	•	•
Cre06g268600t12(CSD1)	•	•	•					•	•	
Cre16g667900t13(AP215)	•	•		•		•			•	•
g8359t1(PHD19)	•		•	•	•	•	•	•	•	
Cre01g034350t13(MYBL13)	•						•		•	•
Cre01g002250t12(GNAT32)		•	•	•		•	•		•	
Cre06g300600t13(SBP8)		•		•	•	•			•	•

	Photosynthesis	Nitrogen	Chlorophyll	Photorespiration	OPPP	Calvin cycle	Carbohydrates	Central metabolism	Amino acids	Lipids
Permanent hubs										
Cre12g558950t13(HMG4)	•	•	•	•	•	•	•	•	•	•
Cre10g453500t12(RWP1)	•	•	•	•	•	•	•	•	•	•
Cre03g149350t13(RWP10)	•	•	•	•	•	•	•	•	•	•
Cre16g679050t12(PHD13)	•	•	•	•	•	•	•		•	•
Cre17g702450t13(SET19)	•	•	•	•	•	•	•		•	•
Cre06g264400t13(Myb3)	•	•	•	•	•	•		•		•
Cre01g051150t13(bZIP13)	•	•	•	•		•	•			
Cre13g566200t12(C3H14)	•	•	•	•		•	•			
g11506t1(MADS2)	•	•	•	•		•	•		•	
Cre03g189650t13(TAZ2)	•	•	•	•		•	•			•
Cre02g079200t12(YB3)	•	•	•				•			
Cre07g321550t12(bZIP2)	•	•	•				•		•	•
g16135t1(MYBL5)	•	•	•						•	
g4485t1(TAZ3)	•	•		•	•	•	•		•	•
Cre02g078700t13(Jumonji3)	•	•		•		•	•			
g17727t1(PHD6)	•	•		•		•	•	•	•	•
Cre02g075650t12(C3H5)	•		•	•		•	•		•	
Cre02g108450t12(MBF1)	•		•	•		•		•	•	
Cre12g520650t12(TUB1)	•		•		•		•	•	•	
Cre06g250950t12(C3H9)	•		•				•	•		
Cre06g266850t12(GATA11)	•		•					•	•	•
Cre17g742700t13(SET13)	•			•	•	•	•		•	•
Cre03g197350t12(MYBL1)	•						•		•	
Cre10g441050t12(PHD17)	•						•			•
Cre03g163200t13(NF-X1)	•								•	•
Cre03g180800t11(Orphans3)		•								

	Photosynthesis	Nitrogen	Chlorophyll	Photorespiration	OPPP	Calvin cycle	Carbohydrates	Central metabolism	Amino acids	Lipids
Cre05g236400t13(GNAT2)		•								
Cre02g082550t12(FHA1)		•				•				
g17767t1(VARL3)		•	•		•			•		•
Cre02g087600t13(SNF26)		•		•		•	•		•	•
g2260t1(SBP22)		•			•		•		•	
Cre07g331450t13(GNAT11)		•			•		•		•	
Cre09g408050t13(mTERF3)		•			•		•		•	
g4645t1(bHLH10)		•					•			
Cre16g653300t12(bZIP14)		•					•		•	•
Cre16g662650t13(GNAT20)		•					•		•	•
g5036t1(C3H2)				•		•				•
g8178t1(Orphans15)							•			
Cre10g446450t12(Orphans7)							•			
g9760t1(VARL1)							•			
Cre06g253250t13(MADS1)									•	
g13222t1(SNF24)										•
Cre10g442850t13(HMG1)										•
g8379t1(Sigma701)										•
Cre12g508050t12(ARID1)										•
Cre12g503450t12(SET16)										•
Cre02g091550t12(Whirly1)										•
Cre16g671900t11(FHA6)										•
Cre16g672300t12(HMG5)										•
Cre03g189850t12(FHA3)										•

Supplementary Figures

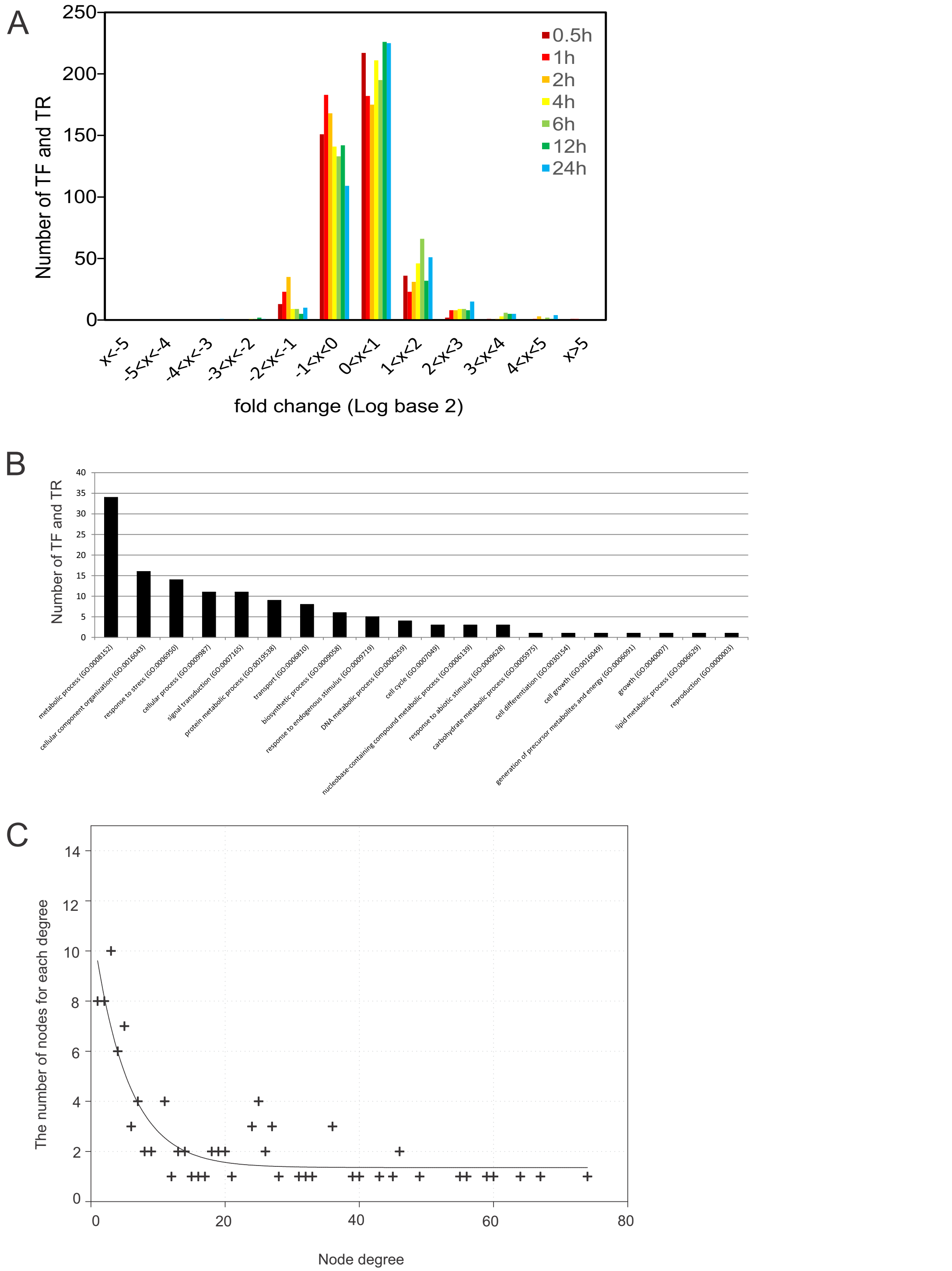

Figure S1.(A) Distribution of changes in transcription factor (TF) and transcriptional regulator (TR) gene expression in cw15 throughout the time course of N deprivation. Values indicate the number of genes that changed by a certain level (log base 2 scale) at any time point during the time course relative to control (time 0). Only the maximum change in expression level per gene was included in the data used to generate this figure. (B) Transcription factor and transcriptional regulator proteins of Chlamydomonas clustered by GO term (biological process category). Functional Class is the GO term and the GO accession numbers are given. Note that most of the transcriptional regulators have been associated with metabolic processes. (C) Node degree distribution of the synergistic regulatory network (10 sub-networks). The X axis is the degree of a node (number of connections between selected TFs and genes of different biological processes), and the Y axis shows the number of nodes that correspond to the degree.

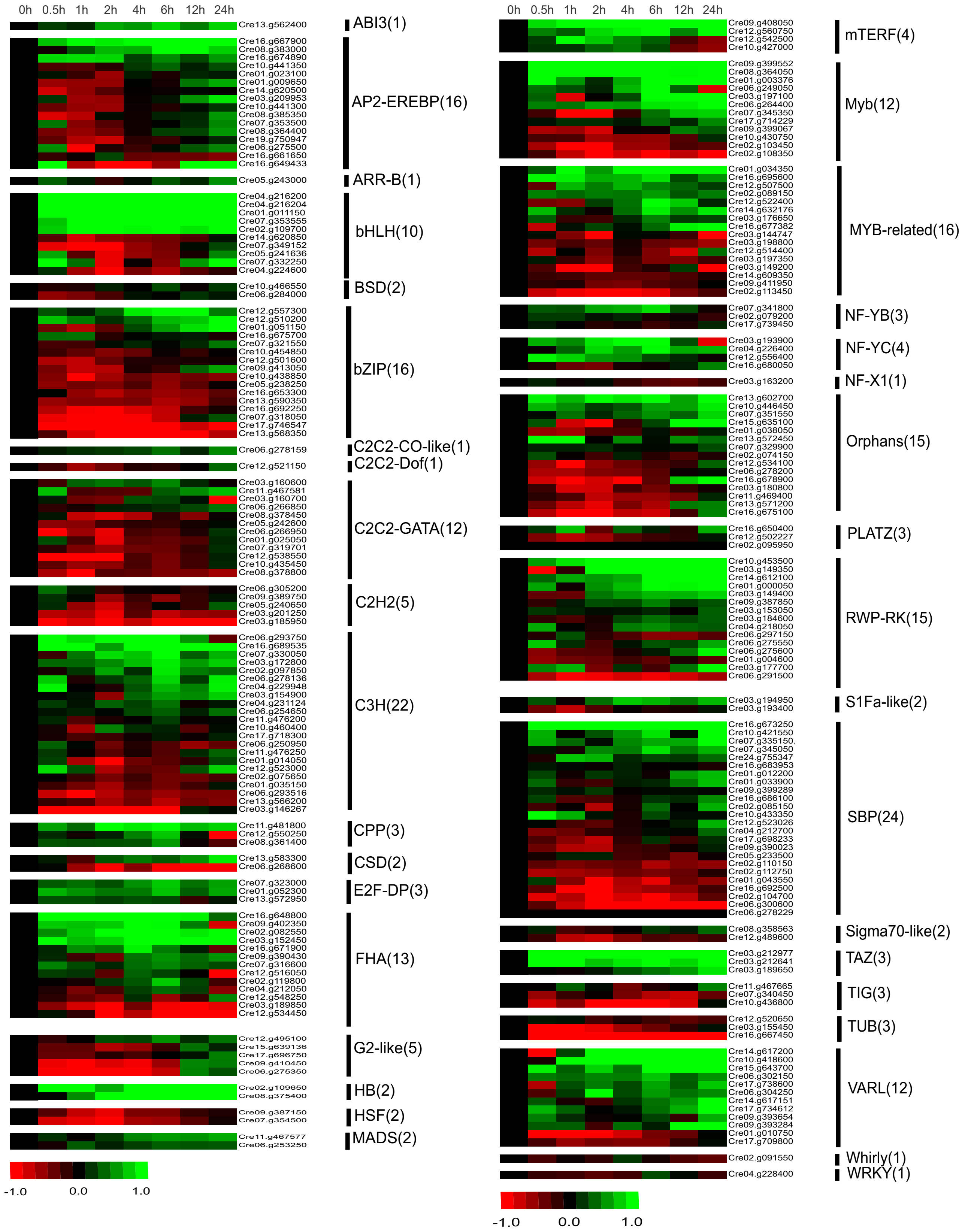

Figure S2. Transcription factor expression profiles in cw15 during the N deprivation time course. Gene expression at different time points is presented as fold change (log base 2 scale) relative to the control. The data were visualized as heat maps with green = up-regulated and red = down-regulated.

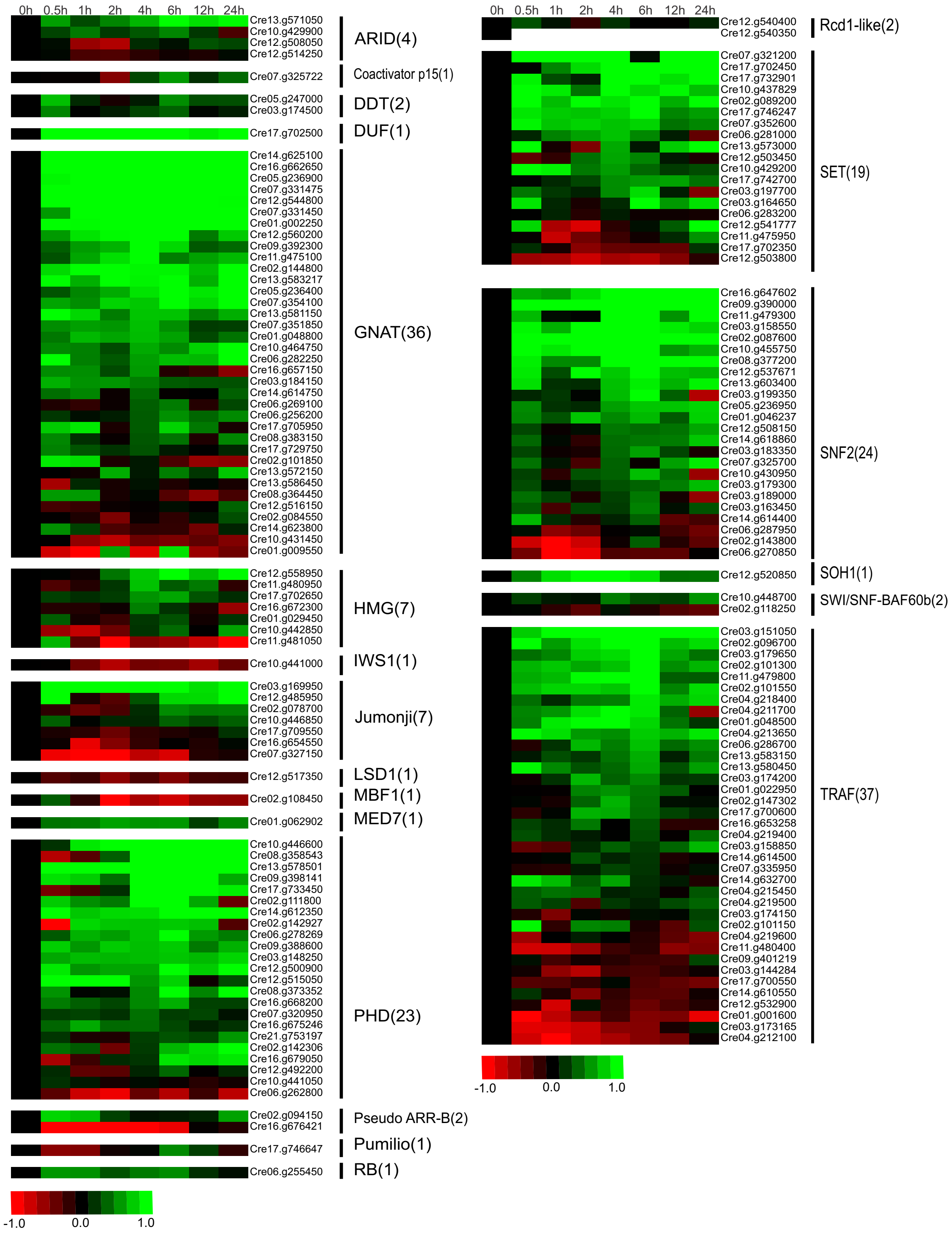

Fig. S3. Transcriptional regulator expression profiles in cw15 during the N deprivation time course. Gene expression at different time points is presented as fold change (log base 2 scale) relative to the control. The data were visualized as heat maps with green = up-regulated and red = down-regulated.

Tests of Normality for the time series of transript data						
0.5h	1h	2h	4h	6h	12h	24h
Shapiro-Wilk's test (P >0.05)						
0.282	.231	0.073	0.041	0.100	0.850	0.642
Histograms						
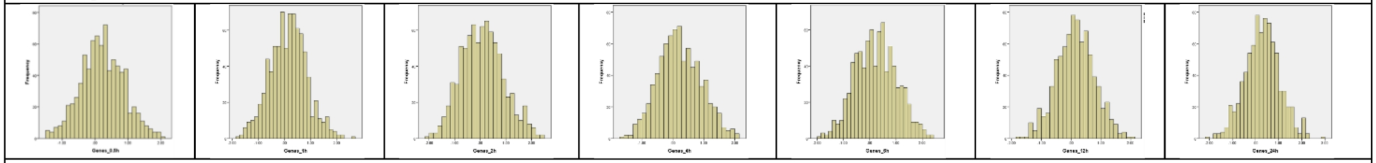						
Normal Q-Q plots						
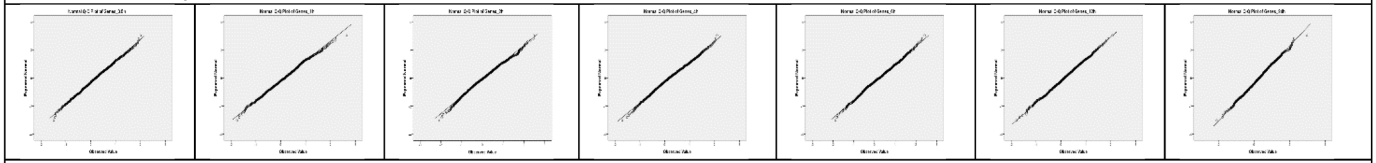						
Box plots						
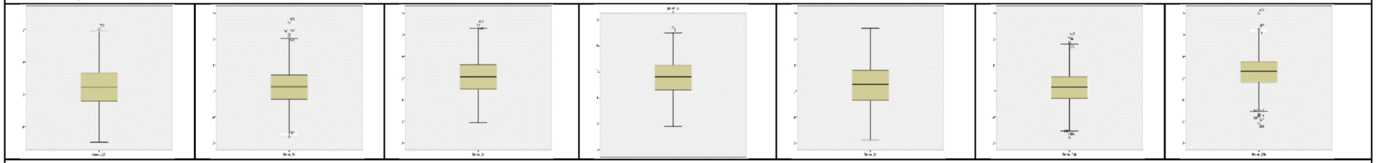						
**Tests of Normality for the time series of metabolite data**						
Shapiro-Wilk's test (P >0.05)						
0.074	0.845	0.041	0.063	0.550	0.346	0.595
Histograms						
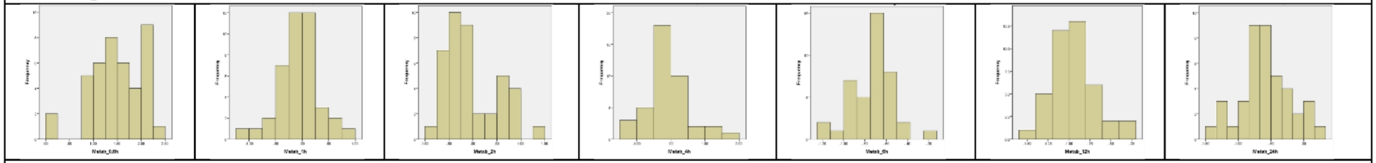						
Normal Q-Q plots						
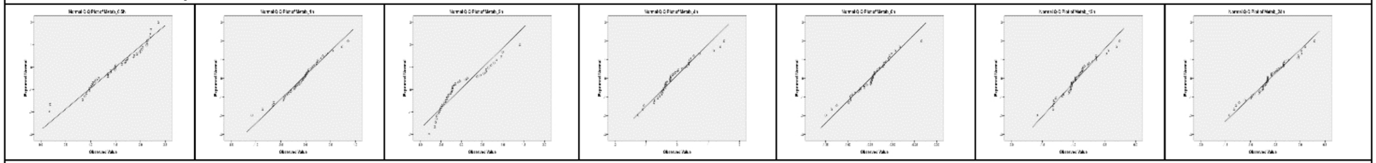						
Box plots						
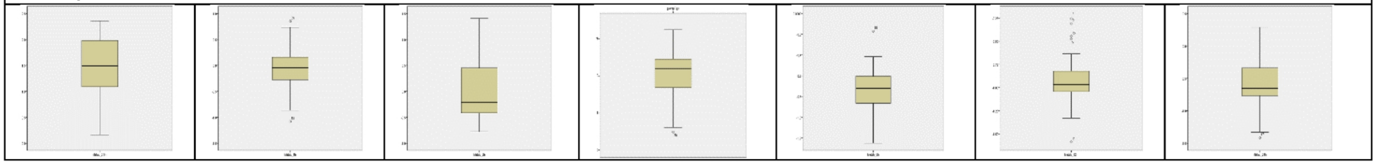						

Fig. S4. Tests of normality for the time series of the transcript and metabolite data. Shapiro-Wilk's values, histograms, normal Q-Q plots and box plots are presented for each time point/each data.

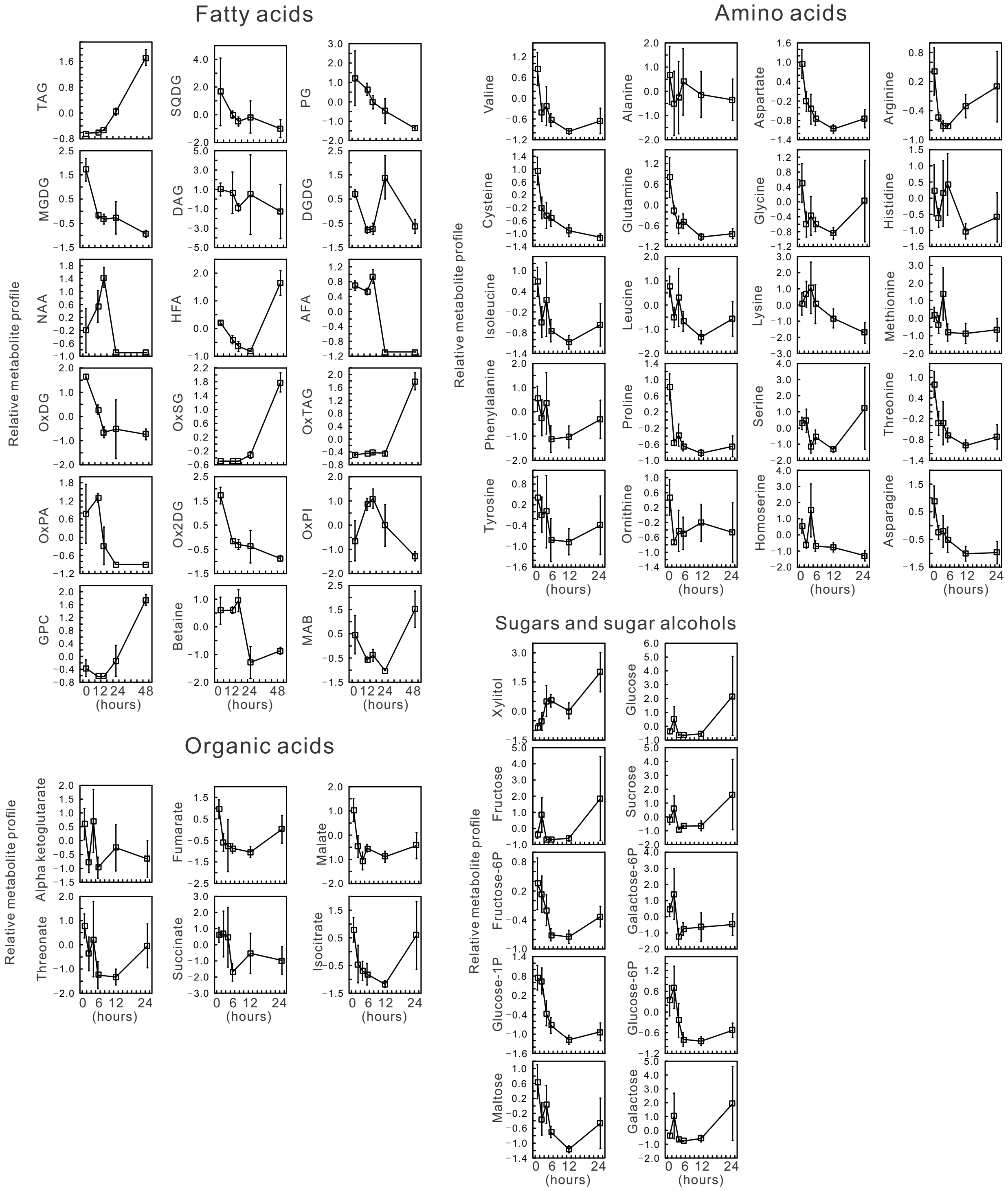

Fig. S5. Primary metabolite profiles in Chlamydomonas during the N deprivation time course. Time points presented for amino acids, organic acids, sugars and sugars alcohol are 0, 0.5, 1, 2, 4, 6, 12 and 24 h. Time points presented for fatty acids are 0, 6, 12, 24, 48 h. Values presented are means of three replicates. OxPI: oxidized phosphatidylinositol; OxDG: oxidized MGDG; OxSG: Acylated sterol glucoside; GPC: 1-alkyl,2-acylglycerophosphocholines; HFA: hydroxy fatty acids; TAG:triacylglycerol; SQDG: sulfoquinovosyldiacylglycerol; PG: phosphatidylglycerolphosphate; OxTAG: oxidized triacylglycerol; OxPA: oxidized phosphatidic acid; NAA: N-acyl amines; MAB: mono-acyl betaine; Ox2DG: oxidized MGDG/MGDG; MGDG: monogalactosyldiacylglycerol; DAG: diacylglycerol; DGDG: digalactosyldiacylglycerol; AFA: amino fatty acids.

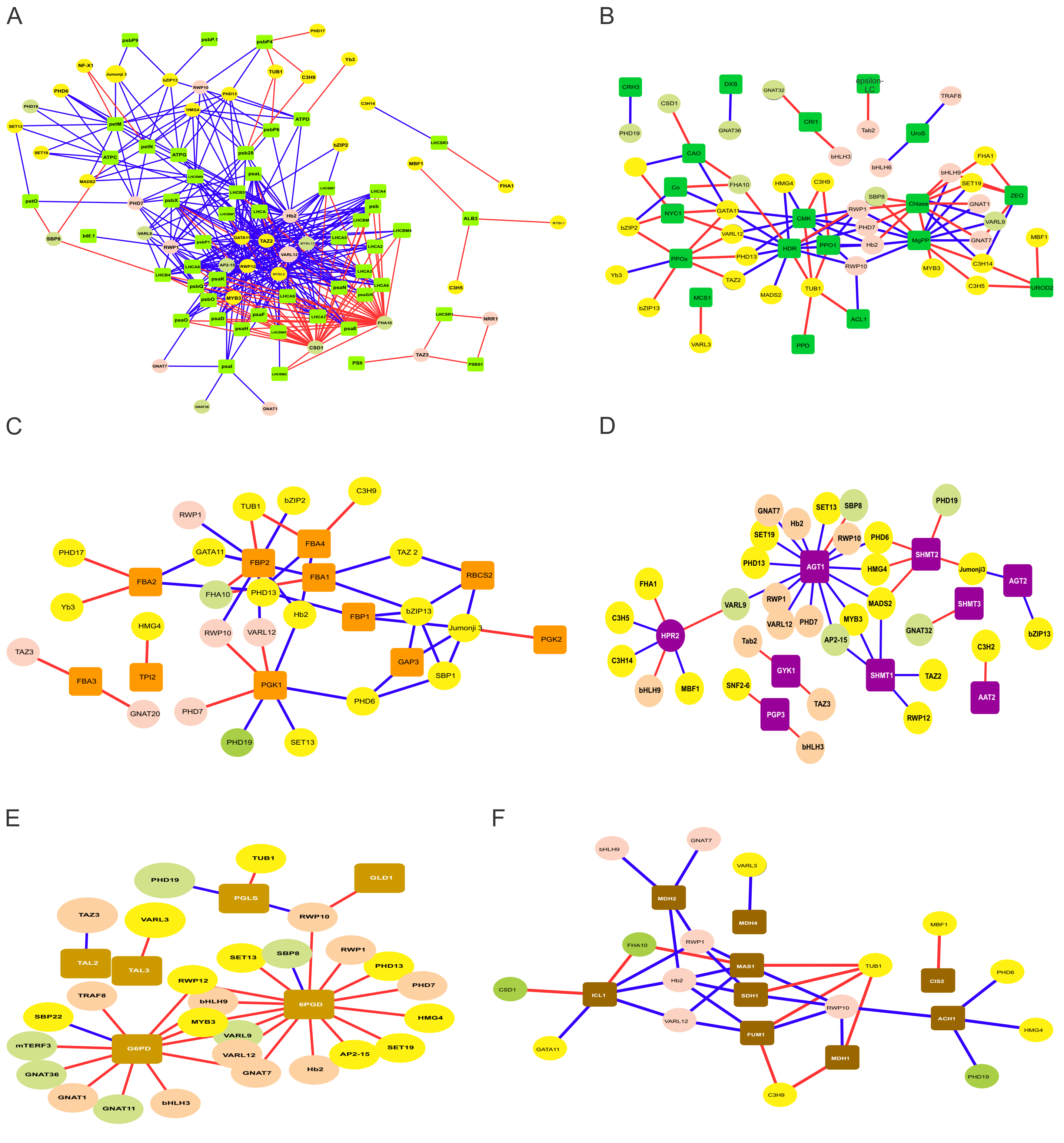

Fig. S6. Cytoscape visualization of correlation networks for different biological processes: (A) photosynthesis, (B) chlorophyll synthesis and degradation, (C) Calvin-Benson-Bassham cycle, (D) photorespiration, (E) oxidative pentose phosphate pathway, (F) citrate and glyoxylate metabolism. Nodes: TFs are represented as red circles for early phase responders, green circles for late phase responders and yellow circles for other pattern of response. Genes for metabolic enzymes are represented as unique color squares specific for each biological process. Lines connecting two nodes represent significant correlations: red represents a positive correlation and blue represents a negative correlation. See Dataset S1 for details on genes included in this analysis.

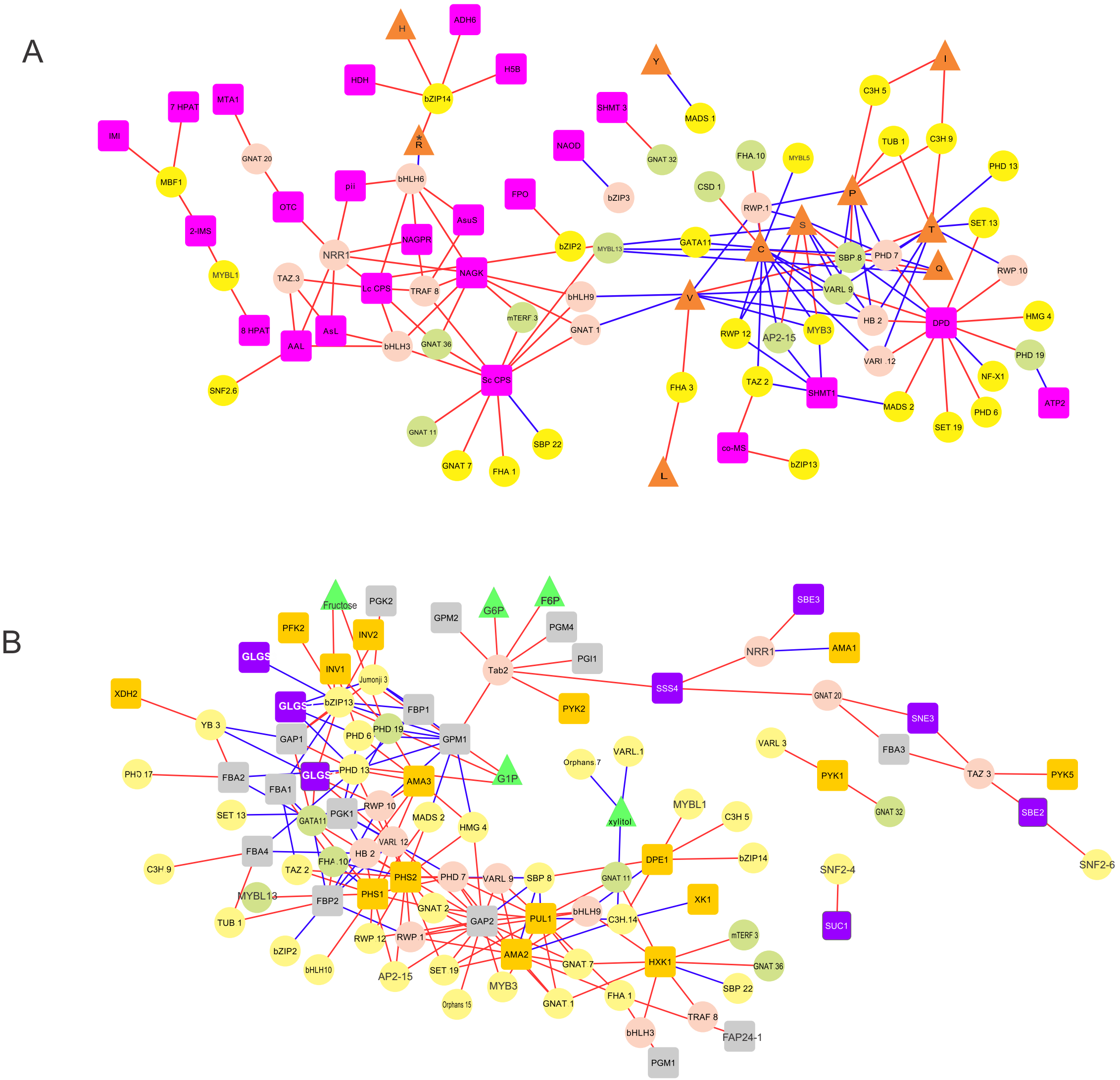

Fig. S7. (A) Visualization of the amino acid biosynthesis regulatory network, based on correlation of a subset of 41 genes with the selected TFs/TRs, in Chlamydomonas during 24 h of N deprivation. Nodes: TFs are represented as red circles for early phase responders, green circles for late phase responders and yellow circles for other pattern of response. Genes for metabolic enzymes are represented as pink squares. Amino acids are represented as orange triangles. Lines connecting two nodes represent significant correlations: red represents positive correlation and blue represents negative correlation. See Table S3 for details on genes included in this analysis.

(B) Visualization of the starch/gluconeognesis/glycolysis regulatory network, based on correlation of a subset of 64 genes and 12 metabolites with the selected TFs/TRs, in Chlamydomonas during 24 h of N deprivation. Genes involved in starch biosynthesis are represented as lilac squares; genes involved in gluconeogenesis are represented as orange squares; genes involved in glycolysis are represented as gray squares. Metabolites are represented as green triangles. Lines connecting two nodes represent significant correlations: red represents a positive correlation and blue represents a negative correlation. See dataset1 for details on genes included in this analysis

A

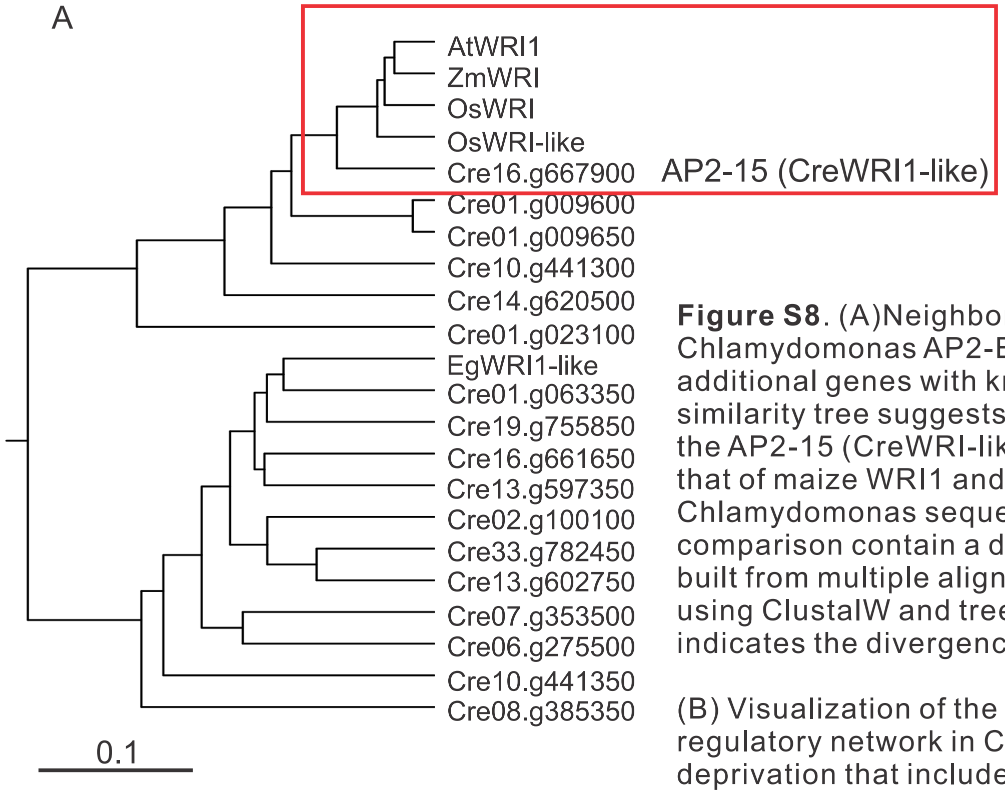

Figure S8. (A)Neighbor-Joining tree of the Chlamydomonas AP2-EREBP TF gene family, with additional genes with known function included. The similarity tree suggests a close relationship between the AP2-15 (CreWRI-like, cre16.g667900) sequence to that of maize WRI1 and the Arabidopsis relative. Other Chlamydomonas sequences included in this comparison contain a double AP2 domain. The tree was built from multiple alignments of protein sequences, using ClustalW and treeView software. The bar indicates the divergence between the sequences.

(B) Visualization of the entire lipid metabolism regulatory network in Chlamydomonas during N deprivation that included 152 lipid metabolism related genes and the 70 TFs/TRs that were differentially expressed. Nodes: TFs/TRs are represented by pink circles (for the early responders) and green circles (for late responders). Metabolism-related genes are represented by olive colored squares. Lines connecting two nodes represent significant correlations: red represents a positive correlation and blue represents a negative correlation. See Table S3 and Fig. 2 for details on genes included in this analysis.

B

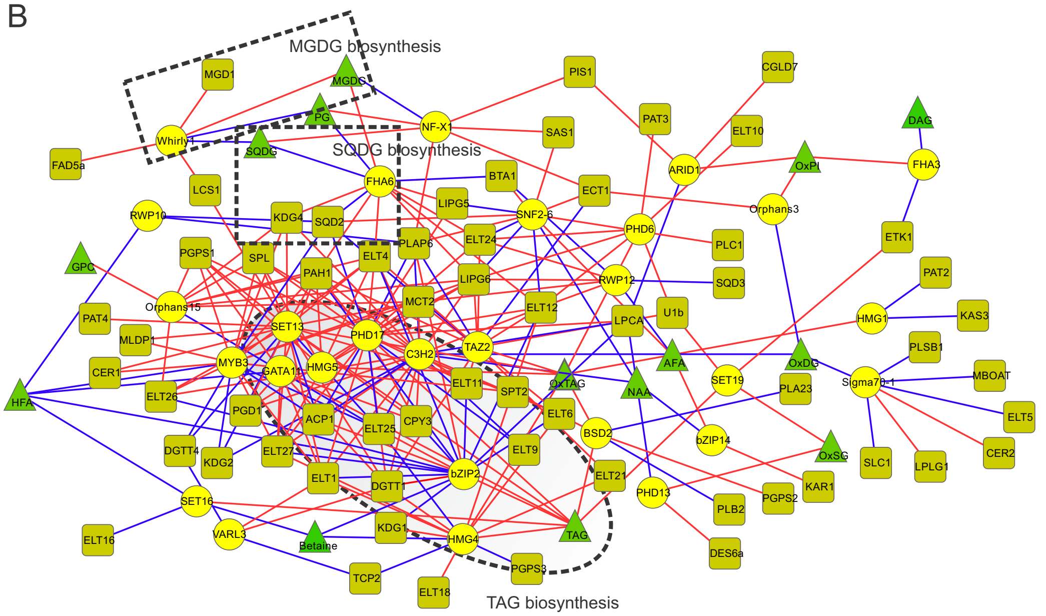

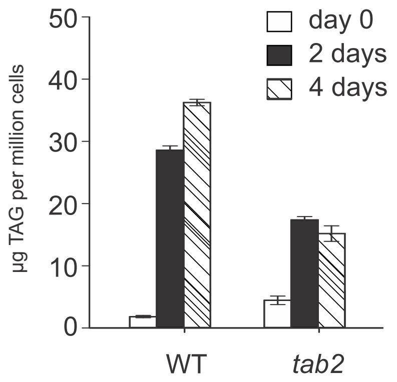

Fig. S9. Changes in TAG content over the time course in the wild type and *tab2* mutant cells grown in N depleted medium. Values are averages of triplicate biological samples, ±SE.

.

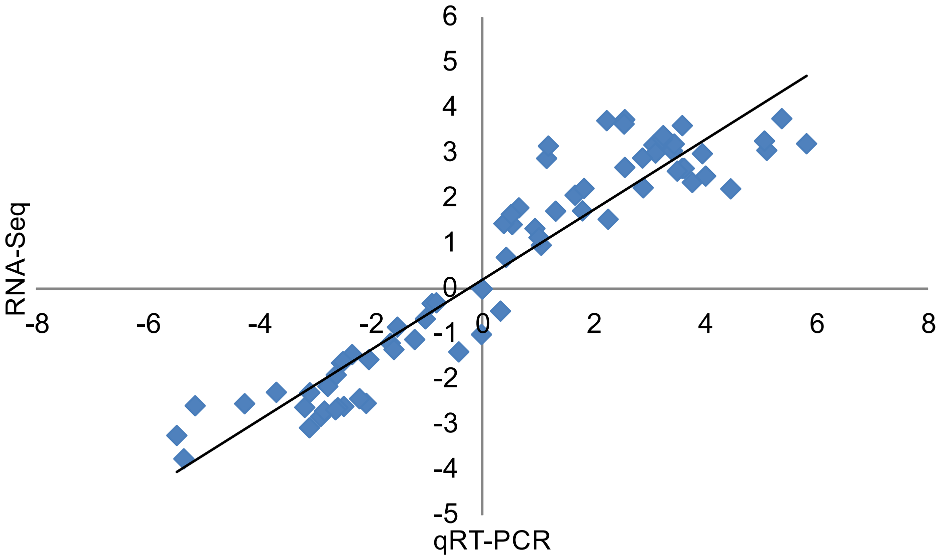

Fig. S10. Verification of gene expression analysis by quantitative real time PCR (qRT-PCR). Individual gene expression ratios (nitrogen deprivation sample to control 0 h sample) were calculated using RPKM data generated by RNA-seq and plotted against calculations performed for the same gene using qRT-PCR.

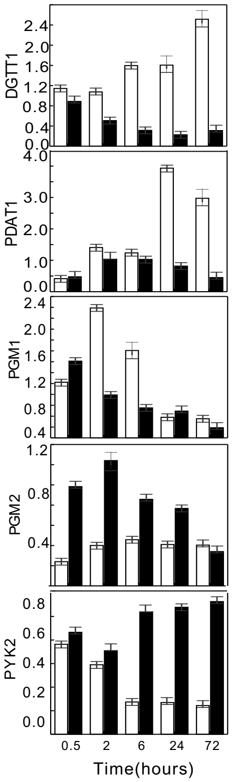

Gene	Forward sequence	Reverse sequence
DGTT1	GCCTTCCTGCTGTACCTCTA	TAAGAGGCCATAACCTTCCA
PDAT1	CGACAGAGTCAAGGACTCAA	GCATACCATTGTTGTCTTGG
PGM1	GCTACGATTACGAGGAGTGC	GGGTCCGTGTACTCAAAGTC
PGM2	AGGCATGTACAAGGACAA GG	AGTCAAAGAAGCCGTACCTG
PYK2	AGGGCGACACCATCTACATC	TTCTTGGCGATGCAGTACAC

Figure S11. Verification of gene expression in WT and *tab2* cells grown in the N deprivation time course by quantitative real-time PCR (qRT-PCR) analysis. The relative transcript levels (within each graph) are represented by white bars for the WT and black bars for the mutant. The time points displayed are 0.5, 2, 6, 24, 72 hours after N depletion. The box to the right contains the sequences of the primers used in the qRT-PCR analysis for each gene.
